# On the Edge of Benefit and Harm: Reactive Oxygen Species in Cancer

**DOI:** 10.3390/ijms27156887

**Published:** 2026-08-01

**Authors:** Anna B. Nikiforova

**Affiliations:** Institute of Theoretical and Experimental Biophysics, Russian Academy of Sciences, Pushchino 142290, Russia; nikiforanna@yandex.ru

**Keywords:** reactive oxygen species (ROS), mitochondria, oxidative stress, redox signaling, carcinogenesis, tumor microenvironment, antioxidant defense, metastasis, apoptosis

## Abstract

Reactive oxygen species (ROS) are central regulators of cancer biology and represent a double-edged target in oncology. At physiological levels, ROS support signal transduction, proliferation, differentiation, and immune responses, whereas sustained ROS imbalance promotes DNA damage, genomic instability, metabolic reprogramming, and remodeling of the tumor microenvironment, thereby contributing to tumor initiation, progression, metastasis, and therapy resistance. Conversely, because many cancer cells operate close to the limit of tolerable oxidative stress, further ROS elevation can trigger apoptosis, ferroptosis, immunogenic cell death, and other cytotoxic programs. This review summarizes the major intracellular and microenvironmental sources of ROS, the mechanisms by which redox signaling shapes malignant transformation and tumor adaptation, and the antioxidant systems that buffer oxidative stress in cancer cells. We further discuss current therapeutic approaches based on both ROS suppression and ROS amplification, including redox-modulating small molecules, radiotherapy, photodynamic and sonodynamic therapy, catalytic nanomaterials, and ROS-responsive prodrugs and drug delivery systems. Particular attention is given to the context-dependent effects of ROS, the antioxidant paradox, tumor heterogeneity, hypoxia, off-target toxicity, and the need for robust redox biomarkers. A deeper understanding of tumor-specific redox vulnerabilities will be essential for developing precise and clinically effective ROS-oriented cancer therapies.

## 1. Introduction

**Figure 1:**
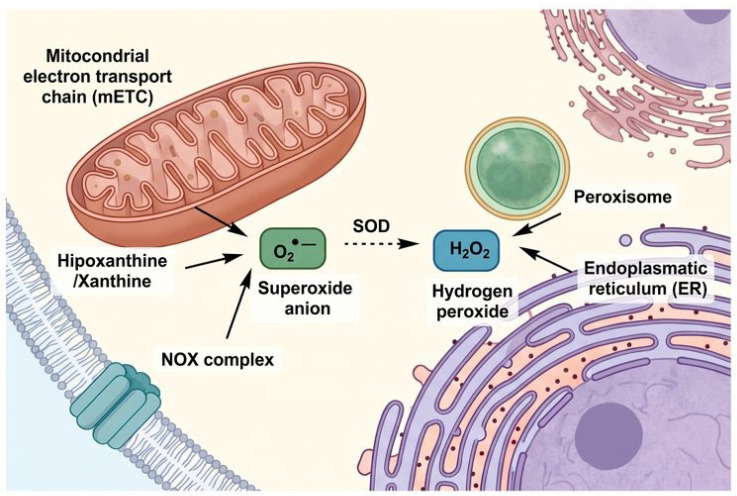
Key sources of ROS in the cell.

Oxygen is a highly reactive molecule capable of giving rise to a wide spectrum of reactive oxygen species (ROS) within the cell. ROS are not merely toxic by-products of aerobic metabolism; rather, they are integral regulators of cellular physiology and participate—often centrally—in metabolism, signal transduction, growth, development, differentiation, and cell death across virtually all cell types [1,2]. Depending on the biological context, ROS may either preserve cellular fitness or drive cells toward distinct forms of death [3,4]. Importantly, virtually every cellular structure and compartment is capable of generating ROS through distinct mechanisms, while an extensive network of antioxidant systems has evolved to prevent excessive ROS accumulation and maintain redox homeostasis [5,6] (Figure 1, Table 1). Carcinogenesis is intimately linked to this redox landscape. On the one hand, elevated ROS levels frequently contribute to the transformation of normal cells into malignant cells; on the other hand, excessive ROS accumulation may overwhelm adaptive defenses and trigger cancer cell death. In the following sections, we discuss the role of ROS at different stages of carcinogenesis and examine how ROS can be both beneficial and detrimental in the context of cancer therapy.

In addition to the classification presented in Table 1, it should be emphasized that the biological effects of ROS are determined not only by their chemical identity but also by their concentration, subcellular localization, duration of exposure, and the presence of transition metal ions and antioxidant systems in the immediate microenvironment. For instance, while O_2_•^−^ is relatively short-lived and poorly membrane-permeable, H_2_O_2_ is stable enough to diffuse across membranes and act as a second messenger in redox signaling. By contrast, •OH reacts at near diffusion-limited rates with virtually all biological macromolecules and is therefore considered the most damaging ROS. ONOO^−^, formed from the rapid interaction of O_2_•^−^ and NO, can nitrate tyrosine residues and inhibit mitochondrial respiration, contributing to both cytotoxic and signaling functions. Understanding these distinctions is fundamental for interpreting the dual roles of ROS in tumor biology and for the rational design of ROS-targeted therapeutic interventions.

## 2. Chemical Bases of ROS Generation and the Role of Transition Metals

It is important to emphasize that many of the reactions responsible for ROS formation and interconversion in the cell are catalyzed by or depend upon transition metal ions, chiefly iron and copper. The most prominent example is the Fenton reaction, in which ferrous iron (Fe^2+^) reacts with hydrogen peroxide (H_2_O_2_) to generate the highly reactive hydroxyl radical (•OH):

Fe^2+^ + H_2_O_2_ → Fe^3+^ + •OH + OH^−^

This process represents one of the primary routes for the production of the most deleterious ROS within the biological milieu and is intimately linked to cellular iron metabolism [7]. Similarly, cuprous ions (Cu^+^) can participate in Fenton-like reactions, generating hydroxyl radicals and promoting lipid peroxidation [8]. Beyond single-step oxidation, transition metals are also engaged in redox cycling with various organic molecules, a process that can markedly amplify ROS production in a catalytic manner. This redox cycling underlies the toxicity of certain chemotherapeutic agents and has been exploited as a therapeutic strategy in the development of novel anticancer compounds [9,10].

## 3. Redox Microcompartmentalization and Signaling Microdomains

A critical aspect of ROS biology that extends beyond the mere identification of ROS sources is the spatial organization of redox signaling. The cellular response to ROS is not determined by a global change in ROS levels but rather by highly localized signaling events occurring within specialized microdomains [11]. This spatial segregation is essential for achieving signaling specificity and for preventing the indiscriminate oxidative damage that would result from unconstrained ROS diffusion.

Mitochondria and the endoplasmic reticulum (ER) form a particularly important redox signaling hub at mitochondria-associated membranes (MAMs), where these two organelles establish close physical contacts. At this interface, ER-derived Ca^2+^ is transferred to mitochondria, where it stimulates oxidative phosphorylation and, under certain conditions, enhances ROS production by the electron transport chain [12]. Reciprocally, mitochondrial ROS can modulate ER calcium homeostasis and ER stress responses, creating a bidirectional signaling loop. MAMs also concentrate redox-sensitive proteins, such as the inositol 1,4,5-trisphosphate receptors (IP3Rs) and voltage-dependent anion channels (VDACs), which are subject to oxidative modification and fine-tune inter-organellar communication [13].

The spatial regulation of ROS is further achieved through the compartmentalized localization of ROS-producing and ROS-scavenging enzymes. For instance, NADPH oxidase 4 (NOX4) is localized primarily to the ER and perinuclear regions, whereas NOX1 and NOX2 are predominantly plasma membrane-associated, generating distinct spatial ROS signatures [14]. Similarly, the localization of superoxide dismutase isoforms (cytosolic SOD1, mitochondrial SOD2, and extracellular SOD3) ensures that superoxide is rapidly converted to H_2_O_2_ in a compartment-specific manner, limiting its diffusion [15].

Hydrogen peroxide, being relatively stable and membrane-permeable, can serve as a diffusible messenger that transmits redox signals between cellular compartments. However, its spatial range is tightly controlled by the local abundance of peroxiredoxins and glutathione peroxidases, which are strategically positioned to create gradients of H_2_O_2_ concentration that encode signaling information [16]. This is elegantly illustrated by the ‘floodgate’ hypothesis, which proposes that localized inactivation of peroxiredoxins allows transient H_2_O_2_ bursts to propagate and oxidize specific downstream targets, achieving signaling specificity even with a relatively stable diffusible messenger [17].

In cancer cells, dysregulation of these spatial control mechanisms contributes to the establishment of a pro-tumorigenic redox environment. For example, aberrant MAM formation and function have been observed in several cancer types, promoting mitochondrial ROS production and sustaining proliferative signaling [18]. Understanding the spatial architecture of redox signaling is therefore essential for developing targeted therapeutic strategies that exploit specific redox microdomains while sparing global redox homeostasis [19].

## 4. Altered ROS Homeostasis in Cancer: Sources and Determinants

**Figure 2:**
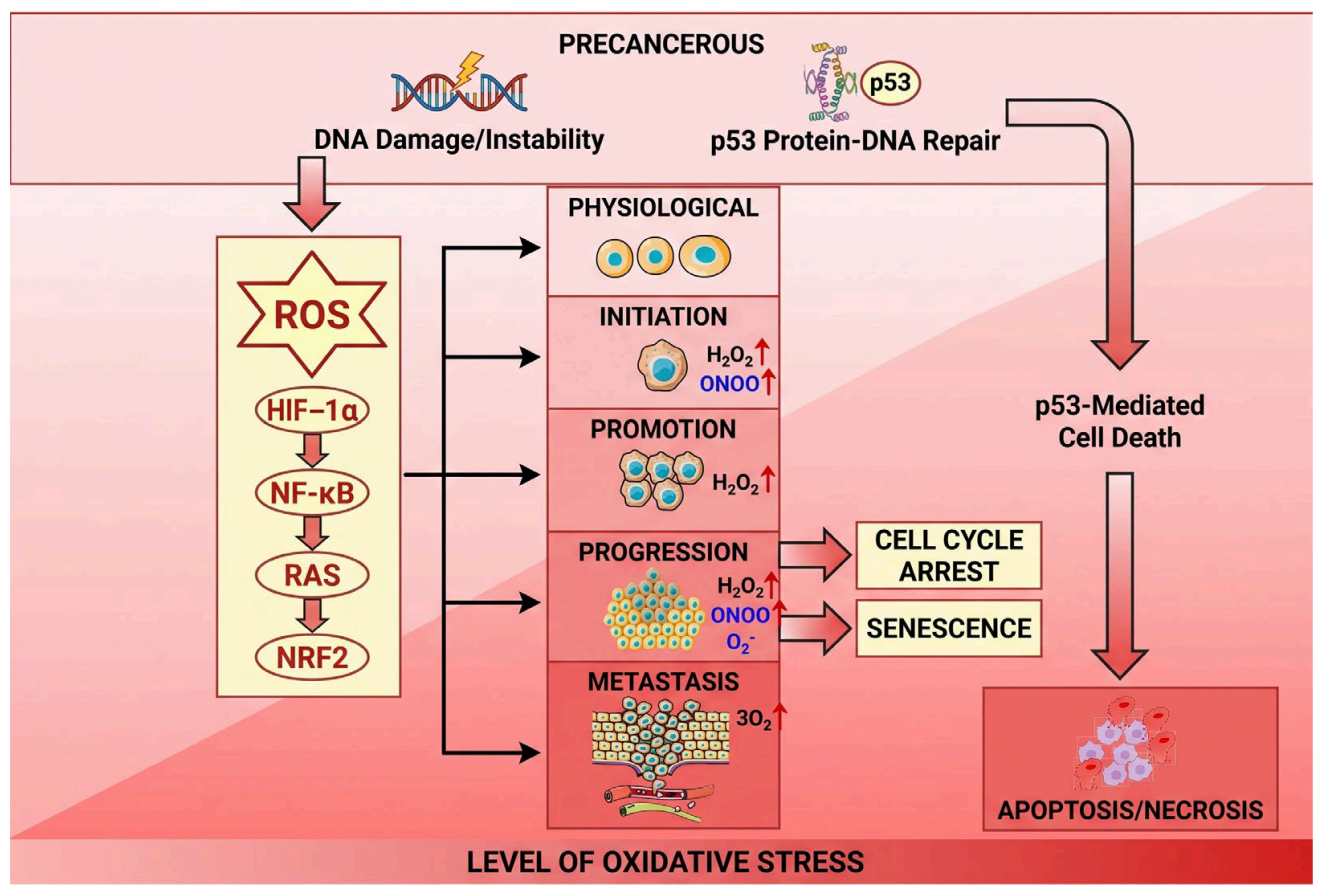
The main stages of carcinogenesis and their relation to oxidative stress.

It is now well established that ROS exert a dual biological role. At low-to-moderate levels—sometimes referred to as beneficial stress or oxidative eustress—ROS function as indispensable mediators of redox signaling and are required for normal cellular physiology [20]. By contrast, excessive ROS production damages key biomolecules, including DNA, membrane lipids, and proteins. A shift in the balance between oxidant generation and antioxidant clearance in favor of oxidants is commonly defined as oxidative stress [21]. Direct quantitative measurement of ROS in vivo remains technically challenging. For example, the detection limit of quantitative electron paramagnetic resonance (EPR) spin trapping is approximately in the nanomolar range, whereas the typically short half-lives of ROS—from microseconds to seconds—severely limit accurate quantification. Consequently, ROS burden is more often inferred indirectly through the detection of oxidative modifications in proteins, lipid peroxidation products, and DNA lesions (Figure 2).

In cancer cells, elevated ROS levels arise from multiple converging sources, including aberrant mitochondrial metabolism and electron leakage from the electron transport chain (ETC), frequently associated with Warburg-like metabolic reprogramming [22,23]; tumor microenvironment (TME)-related factors such as hypoxia, which activates hypoxia-inducible factor 1 (HIF-1) and establishes a positive feedback circuit that further amplifies ROS production [24,25]; inflammatory cytokines secreted by tumor-associated immune cells [26]; and constitutive activation of oncogenic pathways, including NF-κB, NRF2, and PI3K signaling [27,28].

During the initiation phase of carcinogenesis, oxidative stress rises, and the genetic alterations that are selected frequently favor cellular adaptation to this hostile redox environment. Such adaptation is achieved through activation of antioxidant transcriptional programs and/or increased production of nicotinamide adenine dinucleotide phosphate (NADPH), which is required to sustain antioxidant defense systems [29]. ROS and reactive nitrogen species (RNS) not only initiate this adaptive process but also support the expansion of already altered cells during tumor promotion and progression. The initiation stage is characterized by persistent attack by superoxide anion (O_2_•^−^), hydroxyl radical (•OH), peroxynitrite (ONOO^−^), and hydrogen peroxide (H_2_O_2_), ultimately giving rise to the genetic changes required for malignant outgrowth [30]. The subsequent promotion stage is marked by H_2_O_2_-driven proliferation of preneoplastic lesions that have already acquired a degree of growth autonomy [30]. The progression phase, in turn, is characterized by local invasion into adjacent normal tissue and by increasingly complex interactions between malignant cells and stromal or immune components of the TME. At this stage, H_2_O_2_ contributes to epithelial–mesenchymal transition, a prerequisite for metastatic dissemination, and promotes the protumorigenic activity of tumor-associated macrophages that shape the surrounding niche. By contrast, superoxide anion and peroxynitrite are likely exploited by immune cells as cytotoxic mediators against tumor cells [30] (Figure 2).

### The Intratumoral Microbiome as a Source of ROS

An increasingly recognized contributor to the redox landscape of the tumor microenvironment (TME) is the intratumoral microbiome. Recent high-throughput sequencing studies have revealed that diverse bacterial communities reside within tumors across multiple cancer types, including lung, breast, colorectal, pancreatic, and melanoma, often at relatively low biomass but with functional significance. These intratumoral bacteria can influence ROS generation through several distinct mechanisms.

Direct ROS Production: Certain bacterial species, particularly those belonging to the phyla Proteobacteria and Fusobacteria, possess NADPH oxidases and other redox-active enzymes that directly generate superoxide and hydrogen peroxide as by-products of their metabolism [31]. For instance, *Fusobacterium nucleatum*, which is enriched in colorectal tumors, has been shown to produce H_2_O_2_ and induce oxidative DNA damage in adjacent epithelial cells, contributing to genomic instability and tumor progression [32].

Immune-Mediated ROS Generation: Bacterial components, including lipopolysaccharide (LPS), lipoteichoic acid, and flagellin, are recognized by Toll-like receptors (TLRs) and other pattern recognition receptors on immune cells within the TME. This triggers the activation of the NF-κB pathway and induces the expression of inducible nitric oxide synthase (iNOS) and NADPH oxidases in tumor-associated macrophages (TAMs), myeloid-derived suppressor cells (MDSCs), and neutrophils, leading to sustained ROS and reactive nitrogen species (RNS) production [33]. This chronic inflammatory response creates a pro-oxidant microenvironment that supports tumor progression while simultaneously imposing oxidative stress on infiltrating effector immune cells.

Metabolic Reprogramming: The intratumoral microbiome can also influence host cell metabolism, indirectly modulating ROS production. Certain bacterial species produce metabolites such as short-chain fatty acids (SCFAs), hydrogen sulfide (H_2_S), and polyamines that can alter mitochondrial function, enhance glycolysis, and increase NADPH production through the pentose phosphate pathway, thereby shaping the redox capacity of both tumor and stromal cells [34]. For example, bacterial-derived H_2_S has been shown to stimulate mitochondrial biogenesis and enhance mitochondrial ROS production in colorectal cancer cells [35].

Therapeutic Implications: The presence of intratumoral bacteria also has implications for ROS-based therapies. Certain bacterial enzymes, such as nitroreductases and azoreductases, can activate prodrugs that generate ROS specifically within the tumor, a strategy that is being explored in bacterial-directed enzyme prodrug therapy (BDEPT) [36]. Moreover, the intratumoral microbiome composition may influence the efficacy of radiotherapy and photodynamic therapy by modulating the local oxidative environment [37].

Conversely, the tumor redox state itself can shape the intratumoral microbiome. Elevated oxidative stress may select for bacterial species with enhanced antioxidant capabilities, creating a feedback loop that reinforces tumor adaptation and therapy resistance [38]. Understanding the complex interplay between the intratumoral microbiome, ROS, and the TME represents a frontier in cancer biology with significant therapeutic potential.

Because sustained tumor growth depends on a continuous ROS flux, a key question is how cancer cells tolerate persistently elevated protumorigenic ROS while preventing the onset of senescence or apoptosis. In this setting, the upregulation of antioxidant genes is central to adaptation to oncogene-induced oxidative stress [29].

Metastasis involves dissemination of tumor cells through the blood or lymphatic circulation, survival under highly adverse systemic conditions, and subsequent colonization of distant organs. At this stage, the role of antioxidants remains incompletely understood, whereas high ROS levels have been shown to induce apoptosis in a variety of cancer cell types [30].

The data summarized in Table 2 are derived from multiple independent studies employing diverse experimental approaches, including direct measurements of intracellular ROS levels (e.g., using fluorescent probes such as DCFH-DA, MitoSOX, or Amplex Red), assessment of oxidative damage markers (e.g., 8-OHdG, lipid peroxidation products such as MDA and 4-HNE), and analysis of antioxidant enzyme expression and activity. It should be emphasized that ROS levels are highly dynamic and depend on tumor stage, microenvironmental conditions (e.g., hypoxia, pH, nutrient availability, inflammatory infiltrate), and the specific genetic alterations present in a given tumor (e.g., *KEAP1* (Kelch-like ECH-associated protein) mutations, *KRAS* activation, *TP53* status). Therefore, the values presented represent relative estimates rather than absolute quantitative measures. Nevertheless, this classification serves as a useful framework for identifying tumor-specific redox vulnerabilities and for guiding the selection of appropriate ROS-modulating therapeutic strategies.

Regulation of intracellular ROS levels is accomplished by an intricate network of antioxidant enzymes, many of which are metalloproteins. Superoxide dismutases (SODs) constitute the primary line of defense, catalyzing the dismutation of superoxide anion (O_2_•^−^) to hydrogen peroxide (H_2_O_2_). In mammals, three SOD isoforms have been identified: cytosolic Cu/Zn-SOD (SOD1), mitochondrial Mn-SOD (SOD2), and extracellular Cu/Zn-SOD (SOD3), each of which fulfills a distinct role in maintaining redox homeostasis [44]. Catalase, a heme-containing enzyme predominantly localized in peroxisomes, decomposes H_2_O_2_ to water and molecular oxygen, a reaction that is critically important for preventing the formation of the highly toxic hydroxyl radical via Fenton chemistry [45]. The family of heme-containing peroxidases, including glutathione peroxidases (GPXs) and peroxiredoxins (Prxs), also participates in the reduction of H_2_O_2_ and organic hydroperoxides to their corresponding alcohols, utilizing glutathione or thioredoxin as electron donors [46]. In addition to these antioxidant systems, several metalloenzymes contribute to ROS generation. Xanthine oxidase, a molybdoflavoprotein, and mitochondrial cytochrome c oxidase (Complex IV) are notable examples that can serve as sources of superoxide and other ROS under specific pathological or metabolic conditions [47].

## 5. ROS in Early Tumorigenesis

The earliest stages of tumor formation are characterized by oncogenic activation and increased metabolic demand, which together drive enhanced intracellular ROS production. ROS-mediated damage at this stage has been linked to superoxide generation by mitochondria and NADPH oxidases (NOX), as well as to H_2_O_2_ production by 5-lipoxygenase and the endoplasmic reticulum (ER) [23,48].

Several oncogene-dependent mechanisms of ROS generation have been described. First, Ras-driven signaling alters mitochondrial membrane potential and enhances the activity of NADPH oxidases 2 and 4 (NOX2 and NOX4). Second, the anti-apoptotic protein B-cell lymphoma 2 (Bcl-2) can likewise modulate mitochondrial membrane potential and redox output. Third, signal transducer and activator of transcription 3 (STAT3) rewires mitochondrial metabolism and activates NOX4. Fourth, the pleiotropic oncogenic transcription factor MYC perturbs mitochondrial function and biogenesis [23,49]. Cancer cells may also be stimulated to generate ROS in response to tumor necrosis factor-α (TNF-α) secreted by immune cells, while simultaneously being exposed to ROS produced by infiltrating immune populations within the TME. Elevated ROS within cancer cells can perturb MAPK signaling and inhibit redox-sensitive phosphatases such as protein tyrosine phosphatases (PTPs) and phosphatase and tensin homolog (PTEN), thereby reinforcing proliferative and survival pathways [22].

To exploit the proliferative advantage conferred by elevated ROS while avoiding senescence or apoptosis, tumor cells enhance antioxidant defenses and reprogram their metabolism. Evasion of apoptosis despite increased ROS burden requires amplification of the antioxidant machinery, including upregulation of thioredoxin (TXN)- and glutathione (GSH)-dependent enzymes, together with a broader detoxification program [50]. This redox adaptation includes de-repression of the NRF2 pathway through impairment of *KEAP1*-dependent control, resulting in induction of canonical *NRF2* target genes. In parallel, redox remodeling enhances the expression of HIF-responsive genes such as glucose transporter 1 (GLUT1), monocarboxylate transporter 4 (MCT4), and hexokinase 2 (HK2) [51]. Of note, in early tumorigenesis, induction of apoptosis generally requires simultaneous inhibition of both glutathione- and thioredoxin-based antioxidant systems, whereas in premalignant cells selective interference with glutathione synthesis may already be sufficient. This has been demonstrated using agents such as auranofin, sulfasalazine, buthionine sulfoximine, and α-methyl-buthionine sulfoximine [30].

A pivotal regulator of ROS homeostasis in cancer is the transcription factor NRF2, whose stability is controlled by KEAP1, an E3 ubiquitin ligase adaptor that targets NRF2 for proteasomal degradation. Elevated ROS inhibit KEAP1-mediated NRF2 degradation and thereby activate a cytoprotective antioxidant program. NRF2 induces a broad array of antioxidant and detoxifying enzymes, including those required for glutathione biosynthesis via glutamate-cysteine ligase (GCL), which comprises catalytic (GCLC) and modifier (GCLM) subunits [52]. NRF2 is a paradigmatic example of the ambivalent role of antioxidant signaling in cancer. At early stages, NRF2 can suppress tumor formation, as shown in animal models of liver and bladder cancer [53]. At later stages, however, when ROS levels are already markedly elevated, NRF2 often acts as a survival factor that protects cancer cells from ROS-induced death, as reported in lung and pancreatic cancer models [54]. Enhanced NRF2 activation, frequently driven by KEAP1 mutations, has also been observed in several human malignancies [54]. Notably, oncogenes such as KRAS, BRAF, and MYC, which are associated with increased ROS production, also activate compensatory antioxidant programs, including cystine uptake and induction of NRF2-dependent transcription [55].

A critical metabolic adaptation that enables cancer cells to sustain elevated antioxidant capacity is the redirection of glucose flux towards the pentose phosphate pathway (PPP). The PPP serves as the principal source of NADPH, which is indispensable for the regeneration of reduced glutathione (GSH) via glutathione reductase and for the maintenance of thioredoxin in its reduced state through thioredoxin reductase [56]. During early tumorigenesis, oncogenic activation of KRAS, BRAF, or MYC drives the upregulation of glucose-6-phosphate dehydrogenase (G6PD), the rate-limiting enzyme of the oxidative branch of the PPP, thereby enhancing NADPH production to counteract oncogene-induced oxidative stress [57,58]. This metabolic rewiring is further reinforced by the transcription factor NRF2, which directly induces the expression of PPP enzymes including G6PD, 6-phosphogluconate dehydrogenase (PGD), and transketolase (TKT), establishing a positive feedback loop that couples antioxidant defense with anabolic metabolism [59]. Conversely, the tumor suppressor p53 can negatively regulate G6PD activity, thereby limiting NADPH production and promoting ROS accumulation under conditions of metabolic stress [60]. Importantly, the reliance of cancer cells on the PPP for NADPH generation creates a metabolic vulnerability that can be therapeutically exploited, as demonstrated by the selective toxicity of G6PD inhibitors in KRAS-mutant cancers [61].

The tumor suppressor p53 is another critical regulator of ROS in cancer and displays both antioxidant and pro-oxidant functions [62,63]. Through its pro-oxidant activity, p53 can promote ROS accumulation and induce apoptosis or ferroptosis [64]. Conversely, its antioxidant functions may limit the accrual of cellular damage and thus contribute to tumor suppression [65]. Paradoxically, however, by restraining excessive oxidative stress, p53 may in certain settings also support tumor survival by preventing ROS levels from reaching a lethal threshold [66]. Importantly, several cancer-associated point mutations in p53 appear to preserve at least part of its ability to protect cells from ROS-mediated damage [67].

### 5.1. ROS-Mediated Epigenetic Remodeling in Cancer

Beyond classical signaling pathways and transcription factor networks, ROS exert profound effects on the epigenetic landscape, thereby influencing gene expression programs that drive malignant transformation and sustain tumor progression. Oxidative stress modulates DNA methylation patterns through multiple mechanisms. Elevated ROS can directly inhibit the activity of ten-eleven translocation (TET) enzymes, which catalyze the oxidation of 5-methylcytosine (5-mC) to 5-hydroxymethylcytosine (5-hmC), a key step in active DNA demethylation. TET enzymes are iron-dependent dioxygenases that require α-ketoglutarate as a co-substrate and are exquisitely sensitive to oxidative inactivation; ROS-induced depletion of ascorbate and disruption of iron homeostasis further compromise TET activity [68]. Consequently, chronic oxidative stress promotes global DNA hypermethylation at promoter CpG islands, leading to the silencing of tumor suppressor genes, while simultaneously inducing hypomethylation at repetitive elements, thereby promoting genomic instability [69].

Histone modifications are similarly influenced by the cellular redox state. Histone demethylases of the lysine-specific demethylase (LSD) and Jumonji C (JmjC) domain-containing families are themselves redox-sensitive enzymes. The JmjC demethylases, like TET enzymes, are Fe^2+^- and α-ketoglutarate-dependent dioxygenases, rendering them susceptible to oxidative inhibition. Increased ROS levels can therefore promote histone hypermethylation at specific residues, altering chromatin structure and transcriptional accessibility [70]. Additionally, oxidative stress can directly induce post-translational modifications of histones, including carbonylation, nitration, and glycation, which can disrupt nucleosome stability and modulate transcription factor binding [71].

Emerging evidence indicates that ROS-induced epigenetic changes contribute to the establishment of a ‘stress memory’ that enables cancer cells to adapt to fluctuating redox environments. For instance, chronic oxidative stress can drive the recruitment of histone deacetylases (HDACs) to promoters of pro-apoptotic genes, reinforcing their silencing and promoting therapy resistance [72]. Moreover, ROS can modulate the activity of DNA methyltransferases (DNMTs) and histone acetyltransferases (HATs) through direct oxidation of redox-sensitive cysteine residues, further linking the cellular redox state to the epigenetic machinery [73]. These findings highlight the potential of epigenetic therapies—including DNMT inhibitors, HDAC inhibitors, and emerging TET-modulating agents—as combination partners for redox-targeted anticancer strategies [74].

### 5.2. Non-Coding RNAs in Redox Homeostasis

Beyond classical protein-mediated regulation, the cellular redox landscape is finely tuned by non-coding RNAs, including microRNAs (miRNAs) and long non-coding RNAs (lncRNAs), which have emerged as critical regulators of antioxidant responses, ROS production, and oxidative stress signaling in cancer [75].

MicroRNAs modulate redox homeostasis primarily through the post-transcriptional regulation of genes encoding antioxidant enzymes, ROS-generating systems, and redox-sensitive transcription factors. Several miRNAs have been identified as key players in this regulatory network. For instance, miR-200a directly targets the 3’-untranslated region of *KEAP1* mRNA, thereby stabilizing NRF2 and enhancing the antioxidant response in cancer cells [76]. Conversely, miR-144 negatively regulates NRF2 by targeting its mRNA, and its downregulation has been observed in various cancers, contributing to NRF2 hyperactivation [77]. The miR-34 family, which is induced by p53, targets multiple components of the antioxidant machinery, including *SOD2* and *GPX1*, thereby promoting ROS accumulation and contributing to p53-mediated tumor suppression [78]. Additionally, miR-210, a well-known hypoxia-inducible miRNA, modulates mitochondrial metabolism by targeting electron transport chain components, thereby influencing mitochondrial ROS production under hypoxic conditions [79].

Long non-coding RNAs contribute to redox regulation through diverse mechanisms, including chromatin remodeling, transcriptional interference, and microRNA sponging. The lncRNA *NRF2* (also known as *Nrf2-AS1*) has been shown to enhance NRF2 stability by facilitating its interaction with the deubiquitinase USP15, thereby amplifying the antioxidant response in hepatocellular carcinoma [80]. Another lncRNA, *MALAT1*, promotes ROS production by increasing the expression of NOX4 and downregulating antioxidant enzymes, thereby supporting tumor progression and metastasis [81]. The lncRNA *HOTAIR*, which is overexpressed in multiple cancer types, has been implicated in the regulation of ferroptosis through modulation of GPX4 expression, highlighting the intersection of non-coding RNA biology and oxidative cell death pathways [82].

Importantly, the expression of many redox-related non-coding RNAs is itself regulated by ROS, establishing complex feed-forward and feedback loops that integrate redox signals into the broader regulatory architecture of the cell. For example, oxidative stress can activate the transcription of specific lncRNAs that in turn modulate the expression of antioxidant genes, creating a self-perpetuating cycle of redox adaptation [83]. These findings underscore the potential of non-coding RNAs as both biomarkers of oxidative stress and therapeutic targets for redox-based cancer therapy [84].

### 5.3. ROS and Cellular Senescence

The relationship between ROS and cellular senescence represents another dimension of the duality of ROS biology in cancer. Cellular senescence is a state of irreversible cell cycle arrest that can be triggered by various stressors, including persistent oxidative stress and DNA damage [85]. ROS contribute to senescence induction through multiple interconnected mechanisms. Elevated ROS levels cause oxidative DNA lesions, including 8-oxo-7,8-dihydroguanine (8-oxoG) and strand breaks, which activate the DNA damage response (DDR) pathway. Persistent DDR activation, in turn, drives the establishment of the senescence-associated secretory phenotype (SASP) through the activation of p53 and p16^INK4A/pRB pathways [86]. Additionally, ROS can directly induce senescence through telomere dysfunction, as oxidative stress accelerates telomere shortening and promotes telomere uncapping [87].

The role of senescence in cancer is highly context-dependent. In early stages of tumorigenesis, oncogene-induced senescence (OIS) serves as a potent tumor-suppressive barrier that limits the proliferation of cells harboring activating mutations in oncogenes such as KRAS, BRAF, or MYC [88]. OIS is associated with elevated ROS production, and the antioxidant NRF2 has been shown to suppress OIS by limiting ROS accumulation, thereby promoting escape from senescence and malignant transformation [89].

Conversely, in established tumors, therapy-induced senescence (TIS) can be triggered by conventional chemotherapeutics and radiation, contributing to therapeutic efficacy [90]. However, senescent cancer cells and stromal cells that persist within the TME can acquire a SASP characterized by the secretion of pro-inflammatory cytokines, chemokines, growth factors, and matrix-remodeling enzymes. The SASP can promote tumor progression through multiple mechanisms, including paracrine stimulation of proliferation in neighboring cells, induction of epithelial–mesenchymal transition, recruitment of immunosuppressive myeloid cells, and enhancement of angiogenesis [91]. Moreover, senescent cells can produce elevated levels of ROS, creating a pro-oxidant microenvironment that further contributes to genomic instability and supports tumor adaptation [92].

**Figure 3:**
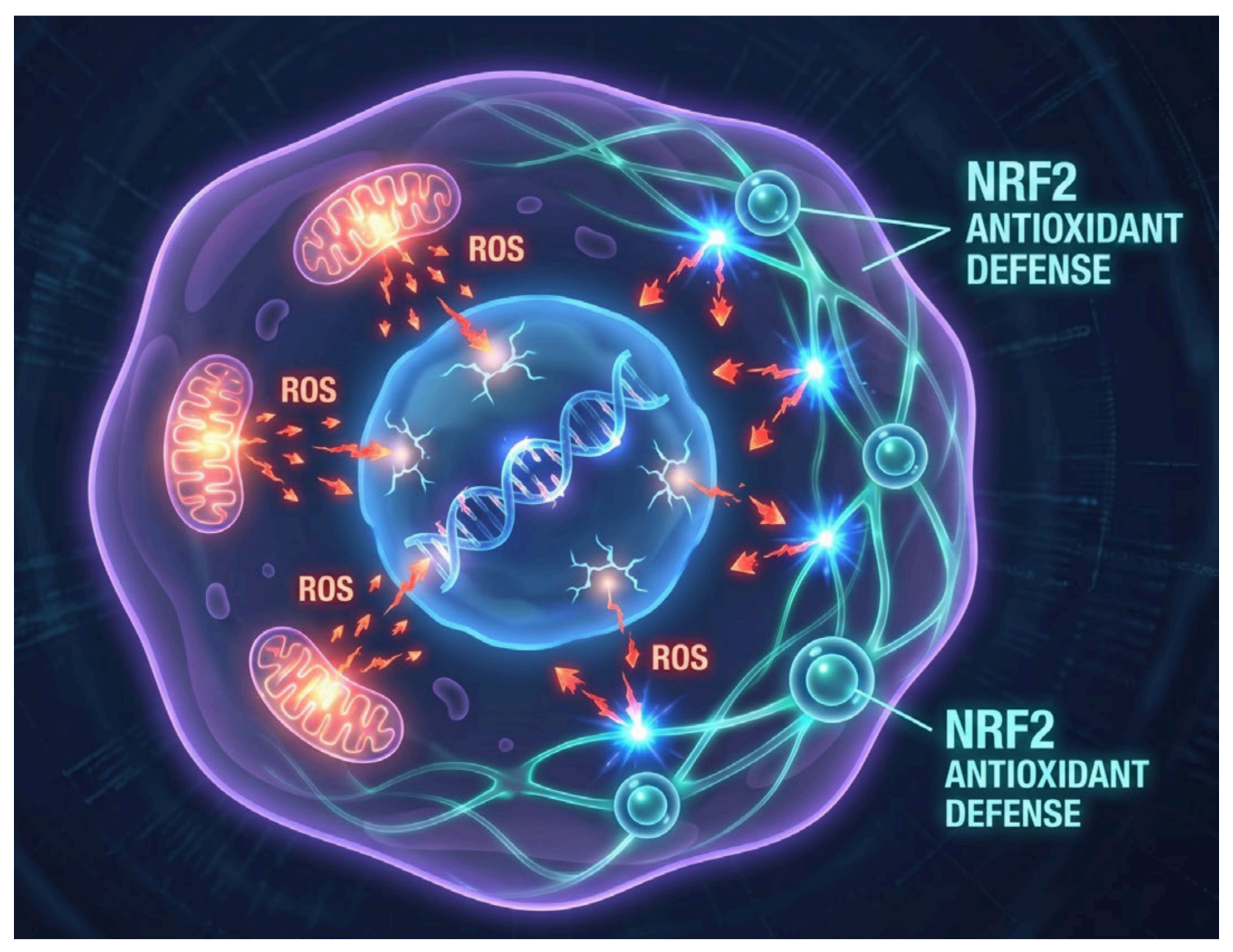
ROS and cancer; early-stage cellular stress and defense.

The emerging concept of ‘senotherapy’—the pharmacological elimination of senescent cells using senolytic agents—has been proposed as a strategy to mitigate the deleterious effects of persistent senescence in the TME. Some senolytics, such as the combination of dasatinib and quercetin, function in part through ROS-mediated mechanisms, highlighting the intersection of senescence, redox biology, and therapeutic intervention [93] (Figure 3).

## 6. ROS and Metastatic Dissemination

Recent evidence indicates that chronic oxidative stress can disrupt neutrophil circadian rhythmicity and promote the formation of neutrophil extracellular traps (NETs) through glucocorticoid release, thereby creating a microenvironment conducive to metastasis [94].

Conversely, a growing body of evidence indicates that excessive ROS can suppress metastatic dissemination. For example, GPX2 knockout increases ROS and inhibits gastric cancer progression and metastasis through disruption of the KYNU–Kyn–AhR signaling axis [95], a pathway known to promote metastatic behavior, as demonstrated in chronic lymphocytic leukemia (CLL) models [96]. Similarly, a one-dimensional Co-PN3 nanozyme loaded with cholesterol oxidase effectively inhibits tumor metastasis by enhancing ROS generation [97].

An unresolved but important paradox is that many solid tumors entering the circulation exhibit limited capacity to establish distant metastases. Human melanoma is particularly notable in this regard because of its typically high metastatic potential. However, melanoma cells in the bloodstream and visceral organs display increased ROS-induced oxidative stress compared with their subcutaneous counterparts, and this is associated with reduced metastatic competence [41]. Strikingly, suppression of oxidative stress with antioxidants increases the ability of melanoma cells to form distant metastases. These findings support the concept that oxidative stress can serve as a major barrier to metastatic colonization [41].

**Figure 4:**
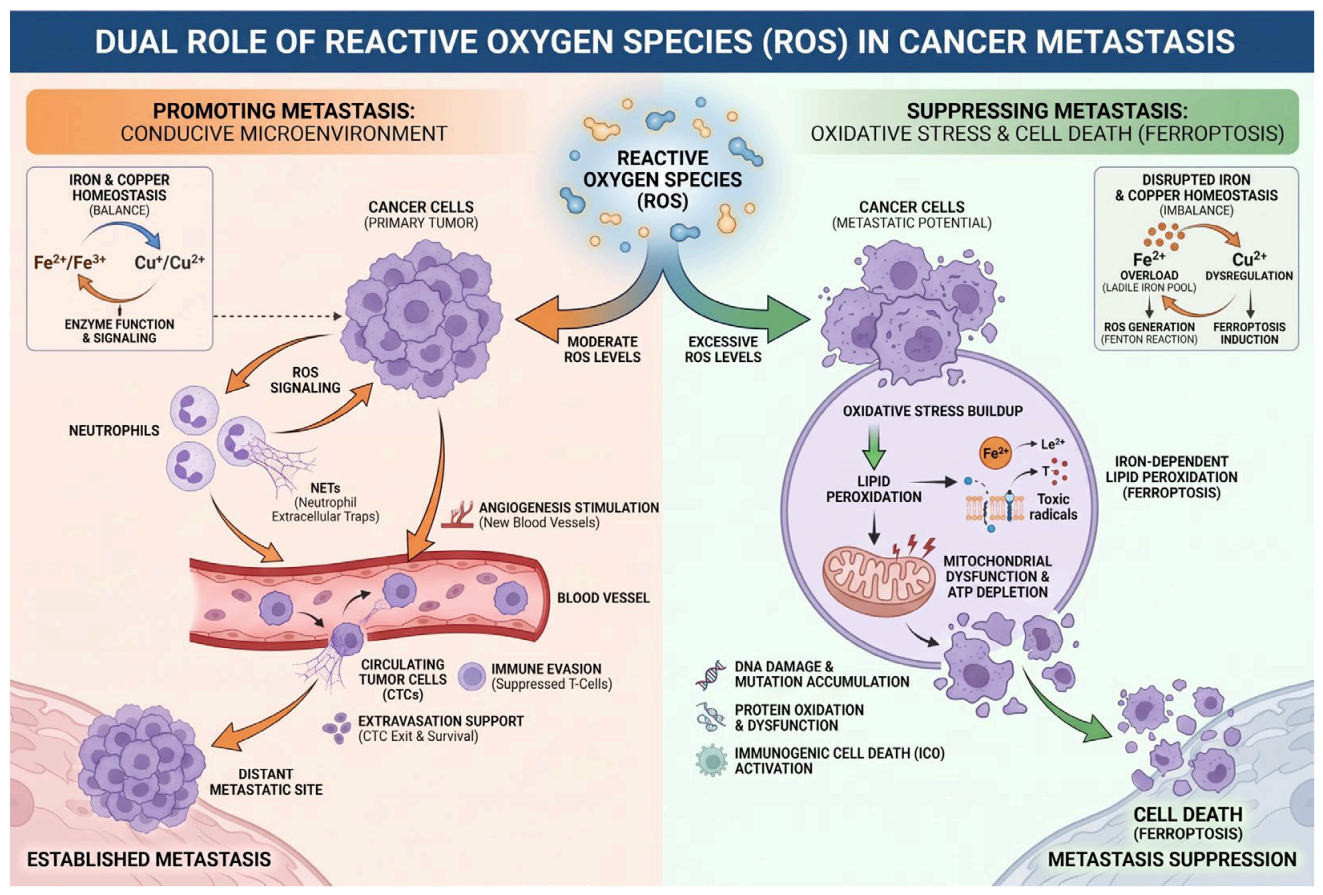
The role of ROS in metastasis.

Iron and copper homeostasis are intimately intertwined with ROS metabolism. Cancer cells frequently exhibit dysregulation of iron metabolism, characterized by elevated levels of intracellular iron (the labile iron pool) and enhanced iron uptake to satisfy their increased metabolic demands [98]. This creates an ‘Achilles heel’ for tumor cells, rendering them more susceptible to the induction of ferroptosis—an iron-dependent form of regulated cell death [99]. Elevated iron levels, in conjunction with enhanced ROS production, promote lipid peroxidation, which constitutes a central event in ferroptosis execution. Similarly, copper, serving as a cofactor for numerous enzymes (e.g., SOD1, cytochrome c oxidase), can also contribute to ROS production via Fenton-like chemistry. However, excess copper is cytotoxic, and cells have evolved sophisticated homeostatic systems, including chaperone proteins (e.g., CCS) and transporters (e.g., ATP7A/B), to tightly control its intracellular levels. Disruption of copper homeostasis can be exploited for therapeutic purposes, for instance, through the use of copper complexes that induce apoptosis or ferroptosis [100] (Figure 4).

## 7. ROS in Cancer Therapy

### 7.1. CAR-T-Cell Therapy and ROS

Chimeric antigen receptor T-cell (CAR-T-cell) therapy is a transformative immunotherapeutic strategy in which a patient’s own T lymphocytes are genetically engineered to recognize and destroy malignant cells. Because these modified cells persist and expand after infusion, CAR-T therapy is often described as a “living drug”. Although CAR-T cells have shown extraordinary efficacy in hematologic malignancies, with response rates approaching 98% in selected settings, their success in solid tumors remains limited, largely because of the profoundly immunosuppressive and pro-oxidant TME [101]. A hallmark of this environment is pathological ROS accumulation, generated by both malignant and stromal cells, which induces DNA damage, mitochondrial dysfunction, and aberrant signaling in CAR-T cells, thereby undermining their antitumor activity [102,103].

Our understanding of ROS in T-cell biology has evolved substantially. Initially viewed solely as toxic by-products of the phagocyte respiratory burst [104], ROS were later recognized as second messengers generated downstream of T-cell receptor (TCR) stimulation, with NOX2 identified as a major enzymatic source [105]. Subsequent work showed that ROS actively shape T-cell fate decisions: effector T cells function in a relatively oxidized intracellular milieu, whereas regulatory T cells maintain a more reduced state [106]. In the context of CAR-T-cell therapy, both ex vivo expansion and the oxidizing milieu of solid tumors impose substantial redox stress, impairing mitochondrial integrity and promoting functional exhaustion [107].

In solid tumors, ROS arise from multiple sources. Tumor cells generate ROS predominantly through electron leakage from mitochondrial ETC complexes I and III, activity of NADPH oxidases (NOX1, NOX2, and NOX4), ER stress and the unfolded protein response, and peroxisomal β-oxidation of fatty acids [108,109]. Warburg-like metabolic rewiring further compromises ETC efficiency and exacerbates electron leakage [110]. In parallel, tumor-infiltrating immune populations—including myeloid-derived suppressor cells (MDSCs), tumor-associated macrophages (TAMs), and regulatory T cells (Tregs)—interact with malignant cells to sustain chronic oxidative stress [111].

Oxidative injury impairs CAR-T-cell activity through several non-mutually exclusive mechanisms. Direct biomolecular damage includes formation of 8-oxoguanine lesions, lipid peroxidation culminating in ferroptosis, and oxidation of key proteins [112,113]. Elevated ROS also activate AMP-activated protein kinase (AMPK), inhibit mTOR and NFAT signaling, suppress c-MYC, and ultimately drive T-cell exhaustion [114]. Moreover, ROS intersect with other immunosuppressive pathways, including hypoxia-associated signaling via the OMA1–OPA1 axis, lactate accumulation, and adenosine signaling, while simultaneously favoring M2 macrophage polarization and Treg stability [115,116].

To overcome these limitations, several engineering strategies have been developed. Genetic approaches include overexpression of antioxidant enzymes such as thioredoxin 1 (TRX1), catalase (CAT), GPX1, and MnSOD, all of which improve CAR-T-cell fitness under pro-oxidant conditions [117,118]. Metabolic reprogramming strategies aim to enhance mitochondrial biogenesis through upregulation of PGC-1α, stimulate fatty acid oxidation, and boost NADPH generation via SIRT3-dependent deacetylation of IDH2 [119,120]. Optimization of CAR design has also been pursued through bicistronic constructs co-expressing catalase (CAR-CAT) or PGC-1α together with the CAR [121]. Pharmacological adjuncts, including N-acetylcysteine (NAC), nicotinamide (NAM), and the NRF2 agonist bardoxolone methyl (CDDO-Me), can reduce ROS accumulation and increase T-cell resilience [122]. In addition, ROS-scavenging nanomaterials and ROS-responsive hydrogels have been developed to directly neutralize extracellular ROS within the TME [123].

Importantly, overcoming oxidative stress in CAR-T-cell therapy does not require complete elimination of ROS, as basal ROS are indispensable for T-cell activation and signaling [124]. Rather, the therapeutic objective is to restore redox balance through precise and tunable modulation. Future progress in this field will likely depend on conditionally activated antioxidant modules, combination strategies capable of targeting multiple TME barriers simultaneously, and rigorous comparative studies designed to identify the most effective engineering paradigm. A deeper understanding of tumor-associated redox landscapes will be essential for the development of next-generation CAR-T-cell therapies for solid tumors.

### 7.2. Therapeutic Exploitation of ROS-Mediated Vulnerabilities

Elevated ROS levels can induce cell cycle arrest, cellular senescence, and cancer cell death [125]. Among the best-characterized mechanisms are activation of the ASK1/JNK and ASK1/p38 signaling axes [126]. In its inactive state, apoptosis signal-regulating kinase 1 (ASK1) is bound to reduced thioredoxin (TRX). Oxidation of TRX by H_2_O_2_ triggers dissociation from ASK1 and subsequent ASK1 activation. This leads to the suppression of anti-apoptotic proteins through the downstream MKK4/MKK7/JNK and MKK3/MKK6/p38 MAPK cascades [127]. Notably, inactivating mutations affecting JNK and p38 signaling have been identified in multiple tumor types, suggesting that therapeutic re-engagement of these pathways may promote tumor cell death [128]. ROS-driven activation of p38 and JNK can also induce cell cycle arrest in malignant cells [129].

Although elevated ROS facilitate tumor initiation and progression, excessive ROS accumulation may paradoxically exert tumor-suppressive effects [39]. Once ROS exceed the buffering capacity of malignant cells, they can inhibit proliferation and trigger apoptosis or necrosis through ER stress, mitochondrial dysfunction, p53-dependent apoptosis, ferroptosis, and other forms of regulated cell death.

Ferroptosis is a distinct form of iron-dependent regulated cell death, which is non-apoptotic in nature and characterized by lethal accumulation of peroxidized membrane lipids [130]. Extensive evidence indicates that ferroptosis plays a key role in elimination of tumor cells and suppression of tumor growth. Cancer cells often display dysregulated iron homeostasis and compensate through increased iron uptake to sustain proliferation, yet this same iron dependency renders them vulnerable to ferroptosis. To withstand chronic oxidative stress, malignant cells upregulate antioxidant systems and lipid metabolic pathways that supply substrates for lipid peroxidation. Glutathione peroxidase 4 (GPX4) is a critical suppressor of ferroptosis because it uniquely reduces lipid hydroperoxides to non-toxic lipid alcohols and thereby prevents propagation of lipid peroxidation. This GPX4-centered protection is a common feature of therapy-resistant cancer states [131]. Pharmacological agents such as erastin and artesunate can promote ferroptosis by functionally disabling GPX4 through interference with reduced glutathione synthesis [132].

The central regulator of ferroptosis is glutathione peroxidase 4 (GPX4), a unique enzyme capable of reducing complex lipid hydroperoxides (LOOH) to their corresponding alcohols (LOH), utilizing glutathione (GSH) as a cofactor [133]. Inactivation of GPX4, whether through depletion of its cofactor GSH (e.g., via inhibition of the cystine/glutamate antiporter system xc^−^ by erastin) or through direct pharmacological inhibition (e.g., by RSL3), results in the accumulation of lipid hydroperoxides. In the presence of ferrous iron (Fe^2+^), these hydroperoxides undergo Fenton-type reactions, generating lipid alkoxyl radicals (LO•) that initiate a chain reaction of lipid peroxidation. This self-propagating process ultimately disrupts membrane integrity and triggers cell death [134]. The frequent dysregulation of iron homeostasis observed in cancer cells renders them particularly susceptible to ferroptosis induction, positioning this pathway as a promising therapeutic target in oncology [135].

Carmustine, an alkylating chemotherapeutic agent marketed as BiCNU, markedly reduces the proliferation and survival of androgen-independent prostate cancer cells when combined with selenite, primarily through disruption of epidermal growth factor receptor (EGFR) signaling [136,137]. This combination induces apoptosis, and its inhibitory effect on EGF-dependent activation of EGFR and its downstream effectors—such as Akt, NF-κB, and ERK1/2—has been attributed mainly to increased ROS production [136].

EGF/EGFR signaling is essential for tumor development [138]. ROS can suppress cancer cell growth by disrupting this axis. When ROS levels exceed the self-regulatory capacity of cancer cells, expression of both EGF and EGFR declines, EGFR phosphorylation is inhibited, and downstream proliferative pathways, including ERK and PI3K/Akt, are attenuated [139].

The pro-oxidant effects of vitamin C have likewise been shown to enhance ROS generation while reducing ERK phosphorylation by limiting EGF release and EGFR activation, ultimately suppressing thyroid cancer cell proliferation [140]. Similarly, koumine, an alkaloid isolated from *Gelsemium elegans*, inhibits proliferation of hepatocellular carcinoma cells [141]. Exogenous H_2_O_2_ has also been shown to inhibit ERK1/2 phosphorylation in breast cancer cells in a dose-dependent manner, thereby reducing proliferation [142].

Copper chaperone for superoxide dismutase (CCS) has emerged as a potential tumor-promoting factor in several malignancies. In breast cancer, CCS is markedly overexpressed and supports both proliferation and migration. Genetic ablation of CCS reduces ERK1/2 phosphorylation, increases ROS production, and consequently suppresses breast cancer cell growth and motility [142].

Oenothein B, a macrocyclic polyphenol, inhibits proliferation of A549 lung cancer cells by inducing apoptosis and arrest at the G1 phase [139]. Mechanistic studies have shown that oenothein B significantly elevates intracellular ROS while upregulating caspase-3, poly(ADP-ribose) polymerase (PARP), Bax, and Bak. Accordingly, its antiproliferative effect is mediated, at least in part, through an ROS-dependent PI3K/Akt/NF-κB signaling pathway [139].

ROS-mediated ER stress also contributes to apoptosis in cancer cells. The ER serves as the principal intracellular Ca^2+^ reservoir, and excessive ROS can trigger Ca^2+^ release from the ER lumen. ER-derived Ca^2+^ reshapes mitochondrial metabolism and modulates the apoptotic threshold during chronic stress. Sustained ROS-induced ER stress activates inositol-requiring enzyme 1 (IRE1), a central ER stress sensor that regulates apoptotic responses in cancer cells [143].

Another major route through which ROS exert anticancer effects is mitochondrial apoptosis [144]. Excessive ROS promote mitochondrial membrane permeabilization, thereby facilitating the release of pro-apoptotic factors—including cytochrome c (Cyt c)—into the cytosol, a defining event of the intrinsic apoptotic pathway. Among the approximately 20 members of the Bcl-2 family, Bax and Bak are the principal executors of mitochondrial outer membrane permeabilization. Upon activation by apoptotic signals, Bax and Bak oligomerize at the outer mitochondrial membrane and drive its permeabilization [40]. Additional pro-apoptotic factors include apoptosis-inducing factor mitochondria-associated 1 (AIFM1) and the inhibitor of apoptosis protein (IAP)-binding mitochondrial protein DIABLO/SMAC [145]. Once released into the cytoplasm, cytochrome c initiates activation of caspase-9 (CASP9), followed by the executioner caspases CASP3 and CASP7 [145].

α-Hederin, a pentacyclic triterpenoid saponin, exhibits anti-inflammatory, antioxidant, antiviral, and anticancer activities across multiple human cancer cell lines [146]. In cancer cells, α-hederin induces excessive ROS accumulation, increases cytosolic cytochrome c, reduces Bcl-2 levels, and elevates Bax, caspase-3, and caspase-9 expression, thereby confirming activation of the mitochondrial apoptotic pathway [147].

Metal-based agents represent another promising class of ROS-centered anticancer therapeutics. In particular, Au(I)–thiourea complexes have attracted significant attention because of their unusual biological properties. These compounds, in which Au atoms are coordinated by sulfur atoms, display pronounced cytotoxicity against multiple tumor cell lines. Their activity is associated with elevated ROS levels, disruption of mitochondrial membrane potential, cytochrome c release, and activation of caspases 9, 7, and 3 [148]. Mechanistically, Au(I)-thiourea complexes downregulate Bcl-2 and upregulate Bax, strongly suggesting ROS-mediated activation of mitochondrial apoptosis [148].

The tumor suppressor TP53, often referred to as the “guardian of the genome”, encodes a multifunctional protein that comprises six major domains and plays a central role in protection against malignant transformation [149]. Beyond its canonical tumor-suppressive role, p53 profoundly influences how both transformed and non-transformed cells respond to chemotherapeutic agents, especially those that induce DNA damage [150]. In response to genotoxic stress, p53 can promote DNA repair, cell cycle arrest, senescence, and apoptosis, thereby limiting transmission of mutations.

Exposure of cells to elevated H_2_O_2_ or other ROS leads to both stabilization of p53 and activation of the DNA damage response. Because DNA damage is a well-established upstream trigger of p53, it has long been assumed that ROS-induced DNA lesions are the principal mechanism underlying p53 activation. However, some ROS—especially H_2_O_2_—also function as bona fide signaling molecules and can activate pathways such as JNK/p38 MAPK independently of overt DNA damage. It is therefore difficult to disentangle the relative contributions of DNA damage, redox signaling, and their interplay in triggering p53 activation [151].

R-goniothalamin, a plant-derived secondary metabolite, displays potent anticancer and pro-apoptotic activity. In breast cancer cells, its cytotoxicity has been linked to induction of oxidative stress and reactivation of mutant p53 [152]. In nude mouse models, administration of R-goniothalamin, either alone or in combination with cisplatin, significantly delayed tumor growth [152].

Piperlongumine (PL), an alkaloid derived from *Piper longum*, has also been shown to induce apoptosis in tumor cells [153]. In the human colon cancer cell lines HT29 and SW620, both of which harbor mutant p53, piperlongumine markedly increased ROS production, protein glutathionylation, NRF2 expression, and expression of p53 target genes such as *BAX* [153]. Because p53 is highly redox-sensitive, it has been proposed that the pro-oxidant milieu induced by piperlongumine promotes functional restoration of mutant p53 through protein glutathionylation, ultimately leading to apoptosis [153].

In addition to organic compounds, redox-active metal complexes have become attractive candidates for anticancer drug development because of their redox reactivity and comparatively moderate toxicity profiles [154]. In hepatocellular carcinoma cells, the Cu(II) complex [Cu(ttpy-tpp)Br_2_]Br, which contains a triphenylphosphine (TPP) ligand, induces marked ROS generation, disruption of mitochondrial membrane potential, mitochondrial Bax aggregation, and cytochrome c release [155]. These findings indicate that the complex triggers apoptosis through the mitochondrial pathway [155]. Mechanistically, ROS-induced translocation of p53 to mitochondria appears to be a critical step in this process [156].

### 7.3. ROS-Activatable Therapeutics and Drug Delivery Systems

Conventional chemotherapy remains limited by poor tumor selectivity and substantial systemic toxicity [157,158,159]. By contrast, malignant tissues typically exhibit higher ROS levels than healthy tissues, creating an opportunity to design ROS-responsive prodrugs and drug delivery systems (DDSs) with improved selectivity [22,138,160].

ROS-responsive moieties used in prodrug design can be broadly classified as cleavable or non-cleavable linkers. Cleavable groups include phenylboronic acid/boronate, which are oxidized by H_2_O_2_ through a Baeyer–Villiger-like rearrangement followed by hydrolysis [161,162]; thioketals, which undergo oxidation to sulfenic acid and subsequent hydrolysis [163,164]; diselenides, which are stepwise oxidized to seleninic acid with concomitant cleavage of the Se–Se bond [165]; aminoacrylates, which undergo singlet oxygen (^1^O_2_)-mediated [2+2] cycloaddition followed by ring collapse [166]; and oxalate esters, which are attacked nucleophilically by H_2_O_2_ [167]. Non-cleavable ROS-responsive groups include thioethers, selenoethers, and telluroethers, which undergo stepwise oxidation and hydrophilic conversion without bond scission [168,169,170]. Importantly, the choice of ROS-responsive group can be tailored to the ROS profile of a specific tumor type: boronate esters are well suited to high-ROS tumors such as glioblastoma and esophageal squamous cell carcinoma, thioketals to intermediate-ROS malignancies such as pancreatic cancer and lung adenocarcinoma, and diselenides to low-ROS tumors such as adrenocortical carcinoma [164,171].

Low-molecular-weight ROS-activatable prodrugs offer several advantages, including defined chemical structure, high drug loading, low molecular mass, and relative ease of toxicological evaluation [172]. Representative examples include a doxorubicin (DOX) conjugate that co-releases H_2_S to mitigate DOX-induced cardiotoxicity [173]; H_2_O_2_-activatable crizotinib prodrugs designed to reduce systemic toxicity [174]; an etoposide prodrug incorporating a coumarin fluorophore for real-time monitoring [175]; a β-lapachone prodrug activated by C–C bond cleavage that exploits NQO1 overexpression in tumor cells [176]; an FK866 (NAMPT inhibitor) prodrug with a coumarin reporter [177]; mitochondria-targeted aminoferrocene-based prodrugs that generate ROS and alter membrane potential [178,179]; a GPX4 inhibitor conjugate with enhanced ferroptosis selectivity [180]; a 10-hydroxycamptothecin prodrug carrying both GSH- and ROS-responsive thioketal and thiamine disulfide linkages [181]; pyrazolopyrimidine prodrugs activated by ROS produced by cold atmospheric plasma [182]; a camptothecin conjugate combining a photosensitizer (TPP-NIR) and a thioketal linker to enable photodynamic activation of mitochondrial apoptosis [183]; a paclitaxel–phthalocyanine conjugate connected through an aminoacrylate linker for far-red-light-triggered photodynamic therapy and site-specific chemotherapy [184]; and a monomethyl auristatin E (MMAE) prodrug activated by hydroxyl radicals generated by external irradiation [185].

Polymeric nanoprodrugs address the unfavorable pharmacokinetics of small-molecule prodrugs by improving tumor accumulation through the enhanced permeability and retention (EPR) effect and active targeting [186,187]. Four major design strategies have been described.

First, ROS-responsive groups can be integrated directly into polymer backbones. Examples include thioether-containing PEG-PPMT copolymers, which become more hydrophilic upon oxidation and release docetaxel (DTX) [188]; selenium-containing block copolymers for co-delivery of cisplatin and paclitaxel with real-time fluorescence monitoring [189]; and boronate-crosslinked α-tocopherol succinate dimers co-assembled with DOX for H_2_O_2_-triggered release [190].

Second, drugs can be tethered to polymers through ROS-labile linkages. Representative systems include mPEG-thioketal-DOX [191]; Lapa@NPs, which co-encapsulate β-lapachone and camptothecin (CPT) and exploit a self-amplifying “ROS generation–drug release” loop [192]; TA-CA- and PTCD@B-based systems designed for self-enhancing release [193]; PEG-TK-DOX/PhA and Ce6@PPE-TK-DOX, which combine photosensitizer-enhanced ROS generation with chemo-photodynamic synergy [194]; and ICG-PBT@NMPs, based on mussel-adhesive protein, which promote tumor penetration and activation of ROS-responsive tirapazamine [195].

Third, stimuli-responsive carriers can encapsulate ROS-responsive low-molecular-weight prodrugs. Examples include lipoic acid-crosslinked nanocapsules (cLANCs) containing disulfide bonds and loaded with Pro-5-FU, in which GSH depletion elevates H_2_O_2_ and accelerates prodrug activation [196]; pH-sensitive micelles co-loaded with β-lapachone and BDOX that enable sequential pH/ROS activation and reversal of multidrug resistance [197]; the supramolecular nanoprodrug HCAG, in which lysosomal acidity disrupts hydrogen bonding and ROS establish a positive feedback activation loop [198]; and PDOX, a polymeric conjugate that requires both acidic pH and elevated ROS/GSH levels for DOX release, thereby creating a dual filtering barrier [199].

Fourth, iron-coordinated nanocarriers can be used to encapsulate low-molecular-weight prodrugs and exploit Fenton chemistry to generate ROS in situ. Examples include PTX-S-DHA co-formulated with PEG2000 and ferrous iron [200]; TKNPDHA-Fc, which combines PEG-thioketal-PTX with dihydroartemisinin (DHA) and ferrocene for simultaneous chemotherapy and ferroptosis induction [201]; and SN38-CA@FC, in which SN38 is linked to cinnamaldehyde via thioacetal and co-assembled with ferrocene to drive lipid peroxidation and GPX4 depletion [202].

In general, metabolites produced from ROS-responsive prodrugs are more hydrophilic and are therefore cleared more efficiently from the body. Thioketal-derived sulfonic acids, for instance, are highly hydrophilic and generally do not cause major organ toxicity [164]. Importantly, the levels of ROS generated therapeutically in these systems are not considered metastasis-promoting. On the contrary, many studies have demonstrated that such ROS-inducing strategies suppress tumor growth by blocking proliferative signaling pathways such as EGF/EGFR and PI3K/Akt, inducing cell cycle arrest, triggering ER stress, activating mitochondrial and p53-dependent apoptosis, and promoting ferroptosis [40,138]. Table 3 discusses prodrugs and their anticancer effects in various cancer models.

**Figure 5:**
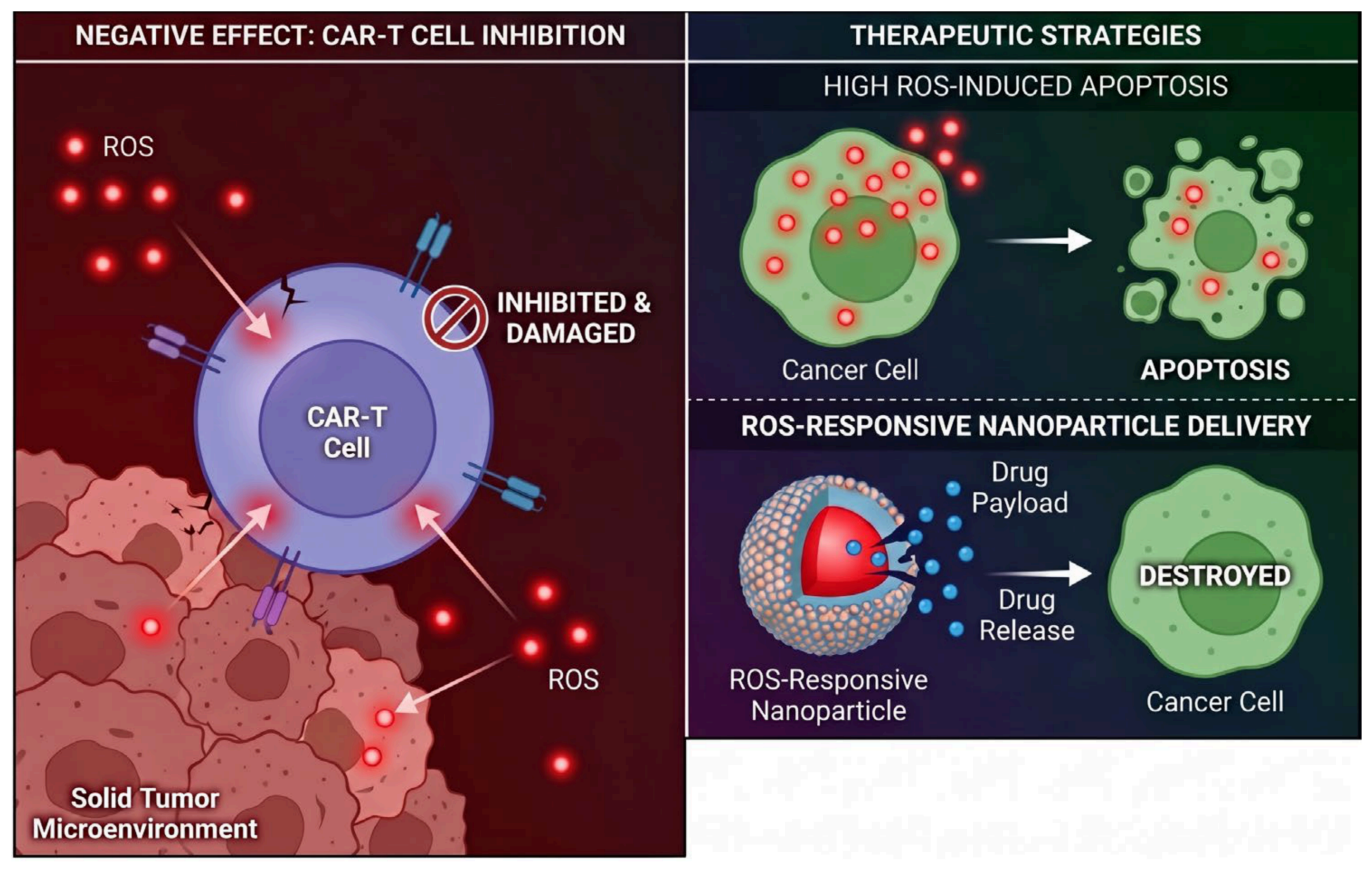
Dual role of ROS in cancer therapy.

Taken together, ROS-responsive DDSs offer precise spatiotemporal control over drug release by exploiting the redox characteristics of the TME. By integrating chemotherapy, immunotherapy, photodynamic therapy, sonodynamic therapy, radiotherapy, and imaging, these systems represent a promising platform for next-generation precision oncology (Figure 5).

## 8. Conclusions and Perspectives

The development of effective ROS-targeted strategies in oncology is fundamentally constrained by the intrinsic duality of ROS biology. In mammalian cells, ROS are indispensable for normal signaling and homeostasis, yet they are also potentially cytotoxic to both malignant and non-malignant tissues. This biological ambivalence makes selective modulation of redox homeostasis exceptionally challenging. One influential framework is the “threshold concept” of anticancer therapy, which proposes that tumor cells—because of their intrinsically elevated ROS levels and compromised redox control—are selectively vulnerable to additional pro-oxidant stress [207]. However, the translational relevance of this concept remains to be firmly established, not least because many studies still lack appropriate healthy cell controls. Large-scale paired clinical studies and carefully designed in vivo experiments are therefore essential.

A more precise definition of the redox threshold that separates adaptive signaling from cytotoxicity requires consideration of several molecular parameters. At the biochemical level, the threshold is fundamentally determined by the redox buffering capacity of the cell, which is largely governed by the glutathione (GSH)/glutathione disulfide (GSSG) ratio and the thioredoxin (Trx)/Trx disulfide (TrxSS) ratio. A shift in these ratios from their physiological values (typically >100:1 for GSH/GSSG) towards a more oxidized state (approaching 10:1 or lower) correlates with the transition from eustress to distress [208]. Quantitative measurements in various cancer cell lines have revealed that an increase in intracellular H_2_O_2_ concentration from basal levels of approximately 0.01–0.1 µM to levels exceeding 1–10 µM can trigger apoptosis or ferroptosis, whereas moderate elevations (0.1–1 µM) are associated with proliferative signaling [209].

Importantly, the threshold is not a fixed value but is dynamically regulated by multiple factors, including the antioxidant enzyme expression profile, the labile iron pool, the lipid composition of cellular membranes, and the rate of mitochondrial respiration [210]. For instance, cells with high GPX4 expression exhibit a higher ferroptosis threshold, whereas cells with elevated polyunsaturated fatty acid (PUFA) content in membrane phospholipids are more susceptible to lipid peroxidation and have a lower threshold for ferroptosis induction [211].

At the molecular level, the transition to cytotoxicity is often governed by the oxidation of specific sensor proteins. The best characterized example is the oxidation of redox-sensitive cysteine residues in KEAP1, which leads to NRF2 stabilization and the induction of a cytoprotective transcriptional program. Saturation of this response—defined by maximal NRF2 target gene expression—sets an upper limit on adaptive protection [212]. Similarly, the oxidation of ASK1-bound thioredoxin triggers the activation of pro-apoptotic MAPK cascades, establishing a switch mechanism that converts a redox signal into a death signal [213].

The threshold can also be modulated by the duration and frequency of ROS exposure. Transient ROS bursts (seconds to minutes) typically elicit adaptive responses, whereas sustained ROS elevation (hours to days) overwhelms antioxidant capacity and drives irreversible damage [11]. This temporal dimension is captured by the concept of ‘hormetic dose–response’, where low-dose or acute ROS exposure is beneficial, while high-dose or chronic exposure is deleterious [214].

From a translational perspective, defining the redox threshold for individual tumors requires the measurement of predictive biomarkers, including the GSH/GSSG ratio, lipid peroxidation products (e.g., malondialdehyde, 4-hydroxynonenal), and the expression levels of GPX4, SOD2, and catalase [215]. Emerging technologies such as genetically encoded redox sensors (e.g., HyPer, Grx1-roGFP) enable real-time monitoring of H_2_O_2_ and GSH/GSSG ratios in living cells and tissues, providing a pathway towards personalized redox-based therapy [216].

Antioxidant-based interventions face equally serious limitations, largely because of the so-called antioxidant paradox. Although antioxidants can protect normal tissues from oxidative damage, tumors may exploit these same mechanisms to support their own survival and progression [40]. Another major issue is the lack of specificity of many currently available redox modulators. For example, NADPH oxidase inhibitors such as diphenyleneiodonium (DPI) and VAS3947 affect more than NOX enzymes alone: DPI interferes with mitochondrial respiratory complexes and nitric oxide synthase, whereas VAS3947 induces apoptosis through alkylation of cysteine thiol groups independently of NOX activity [217]. Such off-target effects can profoundly complicate interpretation of experimental data in studies of tumor redox metabolism. In addition, commonly used compounds such as ascorbate, dimethyl fumarate, and lycopene may act as either pro-oxidants or antioxidants depending on the local microenvironment. For these reasons, antioxidant approaches may be more suitable for chemoprevention than for treatment of established malignancies, where tumors often adaptively strengthen their own defense networks. In this context, deeper analysis of context-dependent effects and of the TME may improve selectivity and help overcome nonspecificity and therapy resistance. As one example, because NADPH oxidase 4 (NOX4) promotes tumor growth and metastasis under hypoxic conditions, selective NOX4 inhibitors may prove useful in hypoxia-driven malignancies [218]. In the absence of highly specific pharmacological tools, genetic strategies such as targeted knockout of relevant redox regulators also deserve consideration.

At the same time, pro-oxidant strategies are themselves limited by major challenges, most notably the metabolic plasticity of cancer cells and the risk of off-target toxicity. Although polypharmacology expands the therapeutic repertoire, true tumor selectivity remains difficult to achieve. The efficacy of pro-oxidant agents is highly dependent on the genetic and metabolic features of a given tumor. A particularly illustrative example is RSL3, a GPX4 inhibitor that induces ferroptosis in pancreatic cancer cells yet shows strikingly different activity depending on KRAS mutational status and the associated metabolic adaptations [219]. These observations underscore the urgent need for context-specific therapeutic regimens that take into account oncogenic drivers, metabolic dependencies, and the redox architecture of the tumor. Reactive metabolites such as H_2_O_2_ may amplify cancer cell death via a bystander effect, but this same property also poses a clear risk to healthy tissues and must therefore be evaluated carefully at both tissue and systemic levels.

Additional complexity arises at the stage of drug development for ROS-dependent signaling pathways. The key challenge lies in the multifaceted, context-dependent, and highly interconnected nature of ROS signaling. Several questions remain central: Is ROS-dependent regulation of a given target evolutionarily conserved? What is the exact molecular mechanism involved? How does this regulation influence specific signaling cascades, and how is it modulated by crosstalk with other pathways? Does the target act within a single signaling route, or does it influence multiple interconnected networks? The TME adds another layer of variability. This is well illustrated in cervical cancer, where MEK inhibition modulates ROS differently across cell lines: ERK suppresses ROS production in C33-A cells but enhances it in SiHa and CaSki cells [220]. Combined inhibition of ROS and ERK produces a synergistic antitumor effect in CaSki and HeLa cells, but not in C33-A or SiHa cells, emphasizing once again the strongly context-dependent relationship between redox signaling and kinase pathways. A precise understanding of ROS–target interactions and pathway dynamics is therefore indispensable for rational therapeutic exploitation with minimal collateral damage.

Modern combination regimens in oncology tend to prioritize ROS induction rather than ROS suppression, largely because of the complex and context-dependent contribution of ROS to malignant transformation and the antioxidant paradox described above. Strategies that intensify oxidative stress in combination with chemotherapy or radiotherapy are especially attractive because they may drive selective ROS hyperaccumulation in tumor cells. Nevertheless, major barriers remain, including limited bioavailability, off-target effects, intratumoral heterogeneity, and the metabolic adaptability of neoplastic tissues [221]. Nanoparticle-based systems offer a particularly promising solution by enabling microenvironment-responsive drug release based on elevated ROS levels, altered pH, or NQO1 overexpression in tumors. Hypoxia, a longstanding obstacle to ROS-oriented therapy, may be partly circumvented by oxygen-independent strategies, including Fenton-type Fe/Cu catalysts, hypoxia-activated prodrugs, sonodynamic therapy, and NQO1-bioactivatable compounds such as β-lapachone [217]. A major translational challenge remains the conversion of mechanistic insights into genuinely effective therapeutic platforms. In this regard, integration of gene editing technologies such as CRISPR or RNA interference with conventional anticancer therapies may offer a particularly powerful approach by enabling precise silencing of genes governing redox homeostasis, such as *NOX4* and *HIF1A*, while retaining the broad cytotoxicity of standard chemotherapeutics.

The antioxidant paradox, which we briefly mention, deserves a more detailed consideration. Its essence lies in the fact that although antioxidants taken in high doses can protect normal cells from oxidative damage, in the context of an existing tumor they can have a pro-oncogenic effect. Cancer cells that have already adapted to increased levels of oxidative stress can use exogenous antioxidants to strengthen their own defense systems, which contributes to their survival, progression, and, as has been shown in some studies, accelerated metastasis [222]. This makes the use of high doses of antioxidants as adjuvant therapy potentially dangerous and requires strict clinical justification [223].

Finally, identification and validation of biomarkers that accurately reflect ROS status will be essential for personalized redox-based therapy. Quantification of oxidative damage products such as F2-isoprostanes and 8-oxo-deoxyguanosine may improve diagnostic sensitivity, although such approaches remain limited by the inherently short-lived and fluctuating nature of ROS themselves [215]. Stable isotope tracing and high-precision analytical platforms, including liquid chromatography–mass spectrometry, may substantially improve detection accuracy, whereas real-time tools such as fluorescence spectroscopy and electrochemical sensors can capture dynamic changes in redox status [224]. Tissue-resolved analyses and the use of specific redox modulators may further refine our understanding of organ- and cell type-specific ROS metabolism. Encouragingly, recent advances in deep-tissue fluorescence imaging, metabolomics, and mass spectrometric mapping already enable in vivo interrogation of tumor redox biology [225]. However, further technological refinement will be required before these approaches can deliver the degree of accuracy and clinical utility needed for routine translational application.

Ultimately, only a comprehensive understanding of how redox modulation influences tumor initiation, progression, metastatic dissemination, and therapy resistance will enable the rational design of context-dependent, clinically meaningful treatment strategies that exploit vulnerabilities in tumor redox homeostasis while minimizing harm to healthy tissues.

## Figures and Tables

**Table 1:** Classification and Types of Reactive Oxygen Species (ROS), Their Biochemical Pathways, and Cellular Targets.

Type of ROS	Chemical Formula	Main Sources of Generation	Key Biochemical Pathways/Enzymes	Main Cellular Targets
Superoxide anion	O_2_•^−^	Mitochondria (ETC complexes I and III), NOX enzymes (NOX1–5), xanthine oxidase, cytochrome P450	One-electron reduction of O_2_; enzymatic activity of NADPH oxidases, xanthine oxidase, and mitochondrial electron leakage	Iron–sulfur clusters, SOD, NO (rapid reaction), proteins with redox-sensitive cysteines
Hydrogen peroxide	H_2_O_2_	Superoxide dismutase (SOD1–3), NOX4, amino acid oxidases, monoamine oxidases, mitochondrial ETC	Dismutation of O_2_•^−^ by SOD; oxidation of flavoproteins; peroxisomal β-oxidation	Peroxiredoxins, glutathione peroxidases, catalase, thiol groups of proteins (cysteine oxidation), DNA (indirectly via Fenton chemistry)
Hydroxyl radical	•OH	Fenton reaction (Fe^2+^ + H_2_O_2_), Haber–Weiss reaction, ionizing radiation (water radiolysis)	Interaction of H_2_O_2_ with transition metal ions (Fe^2+^, Cu^+^); decomposition of peroxynitrite	DNA (base modifications, strand breaks), lipids (lipid peroxidation), proteins (amino acid oxidation)—extremely reactive, indiscriminate damage
Singlet oxygen	^1^O_2_	Photochemical reactions (photodynamic therapy), peroxidase-catalyzed reactions, decomposition of peroxides (e.g., via MPO)	Myeloperoxidase (MPO), catalase, peroxidases; energy transfer from photosensitizers	Unsaturated lipids, DNA (particularly guanine), proteins (histidine, tryptophan, methionine oxidation)
Peroxynitrite	ONOO^−^	Rapid non-enzymatic reaction of O_2_•^−^ with NO	Diffusion-controlled reaction between superoxide and nitric oxide	Protein tyrosine nitration, cysteine oxidation, mitochondrial damage, DNA modification, lipid peroxidation

**Table 2:** Basal ROS Levels, Main Sources, Antioxidant Adaptations, and Redox Signaling Features in Different Tumor Types.

Tumor Type	Relative Basal ROS Level	Main ROS Sources	Key Antioxidant Adaptations	Redox-Sensitive Signaling Pathways	References
Lung cancer (NSCLC, SCLC)	High; highest levels observed in SCLC	Mitochondria, NOX4, hypoxia, cytochrome P450	NRF2-dependent transcription, upregulation of GCLC/GCLM, TXN, SOD2, catalase	PI3K/Akt/mTOR, ERK, constitutive NRF2 activation, HIF-1α (hypoxia-inducible factor)	[30,39]
Breast cancer (including TNBC)	Moderate to high	NOX enzymes (NOX1, NOX2, NOX4), mitochondria, ferroptosis-related pathways	Upregulation of GPX4, glutathione system (GSH/GSSG), TXN system, ferroptosis resistance	MAPK/ERK, PI3K/Akt, STAT3, ferroptosis-dependent pathways, NF-κB	[39,40]
Pancreatic ductal adenocarcinoma (PDAC)	High	KRAS-dependent (NOX4, mitochondria), hypoxia, ER stress	Autophagy, glutaminolysis, enhanced NADPH production via pentose phosphate pathway (PPP), NRF2	KRAS → ERK, PI3K/Akt, NRF2, HIF-1α, YAP/TAZ	[30,39]
Colorectal cancer	Moderate	NOX1 (epithelial expression), mitochondria, inflammation (TNF-α, IL-6)	NRF2, glutathione system, SOD2, catalase, TXN	Wnt/β-catenin, PI3K/Akt, p53-dependent apoptosis, NF-κB	[30]
Hepatocellular carcinoma (HCC)	High	Mitochondria, ER stress, NOX4, cytochrome P450, inflammation	NRF2, elevated GSH, metal metabolism (Fe/Cu homeostasis), upregulation of HO-1	PI3K/Akt/mTOR, MAPK, ferroptosis, NF-κB, STAT3	[30]
Melanoma	High (particularly in metastatic cells)	Mitochondria, NOX2/4, oxidative stress in bloodstream, UV-induced	Upregulation of TXN, GSH, ferroptosis resistance (GPX4), TRX1	MAPK/ERK, PI3K/Akt, NRF2, oxidative stress as a barrier to metastasis	[39,41]
Prostate cancer	Moderate	Androgen-dependent, NOX5, mitochondria, AR signaling	Glutathione and thioredoxin systems, GPX4, elevated GSH	Androgen receptor (AR) signaling, PI3K/Akt, NRF2, NF-κB	[39,42]
Ovarian cancer	High (extracellular: 50–100 µM H_2_O_2_)	Mitochondria, inflammatory microenvironment, NOX enzymes	Upregulation of antioxidant enzymes (SOD, catalase, GPX), metabolic adaptation, enhanced GSH	PI3K/Akt, MAPK/ERK, ferroptosis, NF-κB, STAT3	[43]
Gastric and esophageal cancer	Elevated	Inflammation (TNF-α, IL-1β), NOX enzymes, TME-derived ROS	Immunosuppression via Tregs and effector T cells, upregulation of GSH, NRF2	NF-κB, STAT3, inflammation-associated pathways, PI3K/Akt	[30]
Glioma/Glioblastoma	High	Mitochondria, ER stress, NOX4, hypoxia	NRF2, elevated GSH, metabolic adaptation, upregulation of TXN, SOD2	PI3K/Akt/mTOR, MAPK, survival pathways, HIF-1α	[30,39]

**Table 3:** Examples of ROS-responsive prodrugs and their effect in antitumor therapy.

Name	Key Functional Group	Model	Effect in Tumor Models	Reference
Amonafide	Phenylboronic acid	MDA-MB-231, MCF-10A	Selectively inhibited DNA synthesis	[203]
FK866	Phenylboronic acid	293T, Molt 4, PC-3	An ROS-responsive FK866 prodrug was developed, improving targeting accuracy and therapeutic efficacy	[177]
Etoposide	Phenylboronate	HCT-116 xenografts in BALB/c nude mice	At a dose of 10 mg/kg, the tumor growth inhibition rate reached 46.19%	[175]
Fenretinide	Phenylboronate	HaCaT, A431, SCaBER cells	After CPP treatment, the fenretinide prodrug exhibited increased toxicity in different cell lines	[204]
MMAE	3,5-Dihydroxybenzyl carbamate	4T1	Cell viability in the 4 Gy + 10 nM DHBC-MMAE group decreased to below 30%	[205]
β-Lap	Phenylboronic acid	BALB/c mouse model bearing Mia PaCa-2-induced tumors (NQO1+)	At doses of 20, 40, and 100 mg/kg, tumor inhibition rates were 54.27%, 67.52%, and 71.64%, respectively	[176]
Crizotinib	Phenylboronic acid	H1993, H2228, RUMH	The prodrug showed the greatest activity in H1993 cells, which had the highest ROS levels	[174]
GPX4 inhibitors	Phenylboronate	HT1080, OS-RC-2	Prodrugs of GPX4 inhibitors showed greater ferroptosis selectivity than the parent GPX4 inhibitors	[206]
NAAF	Phenylboronate	BL-2, A2780, DU-145, Jurkat, HDF	The NAAF prodrug demonstrated greater selectivity toward cancer cells, with milder effects on normal cells	[178]

## Data Availability

No new data were created or analyzed in this study. Data sharing is not applicable to this article.

## Abbreviations

The following abbreviations are used in this manuscript:

AIFM1	apoptosis-inducing factor mitochondria-associated 1
AhR	aryl hydrocarbon receptor
AMPK	AMP-activated protein kinase
ASK1	apoptosis signal-regulating kinase 1
BAK	Bcl-2 homologous antagonist/killer
BAX	Bcl-2-associated X protein
Bcl-2	B-cell lymphoma 2
BRAF	B-Raf proto-oncogene, serine/threonine kinase
CAP	cold atmospheric plasma
CAR	chimeric antigen receptor
CAR-T	chimeric antigen receptor T cell
CASP3,7,9	caspase 3, 7, 9
CAT	catalase
CCS	copper chaperone for superoxide dismutase
CLL	chronic lymphocytic leukemia
CPT	camptothecin
Cytc	cytochrome c
DDS	drug delivery system
DHA	dihydroartemisinin
DPI	diphenyleneiodonium
EGF	epidermal growth factor
EGFR	epidermal growth factor receptor
EPR	electron paramagnetic resonance
ER	endoplasmic reticulum
ERK	extracellular signal-regulated kinase
ETC	electron transport chain
GCL	glutamate-cysteine ligase
GCLC	glutamate-cysteine ligase catalytic subunit
GCLM	glutamate-cysteine ligase modifier subunit
GLUT1	glucose transporter 1
GPX1,2,4	glutathione peroxidase 1, 2,4
GSH	glutathione
HIF	hypoxia-inducible factor
HK2	hexokinase 2
H_2_O_2_	hydrogen peroxide
H_2_S	hydrogen sulfide
IAP	inhibitor of apoptosis protein
KEAP1	Kelch-like ECH-associated protein 1
LC–MS	liquid chromatography–mass spectrometry
MAPK	mitogen-activated protein kinase
MCT4	monocarboxylate transporter 4
MDSC	myeloid-derived suppressor cell
MEK	mitogen-activated protein kinase
MnSOD	manganese superoxide dismutase
mTOR	mechanistic target of rapamycin
NADPH	reduced nicotinamide adenine dinucleotide phosphate
NAM	nicotinamide
NAMPT	nicotinamide phosphoribosyltransferase
NET	neutrophil extracellular trap
NFAT	nuclear factor of activated T cells
NF-κB	nuclear factor kappa B
NOX	NADPH oxidase
NQO1	NAD(P)H quinone dehydrogenase 1
NRF2	nuclear factor erythroid 2-related factor 2
OGG1	8-oxoguanine DNA glycosylase 1
ONOO^−^	peroxynitrite
O_2_•^−^	superoxide anion
•OH	hydroxyl radical
PARP	poly(ADP-ribose) polymerase
PDT	photodynamic therapy
PTEN	phosphatase and tensin homolog
RNS	reactive nitrogen species
ROS	reactive oxygen species
SIRT3	sirtuin 3
STAT3	signal transducer and activator of transcription 3
TAM	tumor-associated macrophage
TCR	T-cell receptor
TME	tumor microenvironment
TNF-α	tumor necrosis factor alpha
TP53	tumor protein p53
TRX1	thioredoxin 1
TXN	thioredoxin
^1^O_2_	singlet oxygen

## References

[1] Balaban R.S., Nemoto S., Finkel T. (2005). Mitochondria, Oxidants, and Aging. Cell.

[2] Zuo J., Zhang Z., Luo M., Zhou L., Nice E.C., Zhang W., Wang C., Huang C. (2022). Redox signaling at the crossroads of human health and disease. MedComm.

[3] Green D.R., Reed J.C. (1998). Mitochondria and Apoptosis. Science.

[4] Cho Y.S., Challa S., Moquin D., Genga R., Ray T.D., Guildford M., Chan F.K. (2009). Phosphorylation-Driven Assembly of the RIP1-RIP3 Complex Regulates Programmed Necrosis and Virus-Induced Inflammation. Cell.

[5] Loew O. (1900). A New Enzyme of General Occurrence in Organisms. Science.

[6] Grosche J., Meißner J., Eble J.A. (2018). More than a syllable in fib-ROS-is: The role of ROS on the fibrotic extracellular matrix and on cellular contacts. Mol. Asp. Med..

[7] Prousek J. (2007). Fenton chemistry in biology and medicine. Pure Appl. Chem..

[8] Jomova K., Valko M. (2011). Advances in metal-induced oxidative stress and human disease. Toxicology.

[9] Zhang P., Sadler P.J. (2022). Redox-active metal complexes for cancer therapy. Chem. Soc. Rev..

[10] Valko M., Jomova K., Rhodes C.J., Kuča K., Musílek K. (2022). Redox and non-redox metal-induced formation of free radicals and their role in human disease: An update. Arch. Toxicol..

[11] Sies H., Jones D.P. (2020). Reactive oxygen species (ROS) as pleiotropic physiological signalling agents. Nat. Rev. Mol. Cell Biol..

[12] Marchi S., Patergnani S., Missiroli S., Morciano G., Rimessi A., Wieckowski M.R., Giorgi C., Pinton P. (2021). Mitochondrial and endoplasmic reticulum calcium homeostasis and cell death. Cell Calcium.

[13] Perrone M., Caroccia N., Genovese I., Missiroli S., Modesti L., Pedriali G., Vezzani B., Vitto V.A.M., Antenori M., Lebiedzinska-Arciszewska M. (2020). The role of mitochondria-associated membranes in cellular homeostasis and diseases. Int. Rev. Cell Mol. Biol..

[14] Panday A., Sahoo M.K., Osorio D., Batra S. (2015). NADPH oxidases: An overview from structure to innate immunity-associated pathologies. Cell Mol. Immunol..

[15] Zhou C., Lyu L.H., Miao H.K., Bahr T., Zhang Q.Y., Liang T., Zhou H.B., Chen G.R., Bai Y. (2020). Redox regulation by SOD2 modulates colorectal cancer tumorigenesis through AMPK-mediated energy metabolism. Mol. Carcinog..

[16] Lismont C., Revenco I., Fransen M. (2019). Peroxisomal Hydrogen Peroxide Metabolism and Signaling in Health and Disease. Int. J. Mol. Sci..

[17] Thapa P., Jiang H., Ding N., Hao Y., Alshahrani A., Wei Q. (2023). The Role of Peroxiredoxins in Cancer Development. Biology.

[18] Zhu Y., Tang G., Tang M., Zhou B., Xu J., Ma X., Li Z., Liu G., Han Y. (2026). The role of mitochondria-associated membranes in tumorigenesis. Discov. Onc.

[19] Verma P., Rishi B., George N.G., Kushwaha N., Dhandha H., Kaur M., Jain A., Jain A., Chaudhry S., Singh A. (2023). Recent advances and future directions in etiopathogenesis and mechanisms of reactive oxygen species in cancer treatment. Pathol. Oncol. Res..

[20] Azzi A. (2022). Oxidative stress: What is it? can it be measured? where is it located? can it be good or bad? can it be prevented? can it be cured?. Antioxidants.

[21] Sies H. (2023). Oxidative eustress: The physiological role of oxidants. Sci. China Life Sci..

[22] Moloney J.N., Cotter T.G. (2018). ROS signalling in the biology of cancer. Semin. Cell Dev. Biol..

[23] Raimondi V., Ciccarese F., Ciminale V. (2020). Oncogenic pathways and the electron transport chain: A dangerous ROS liaison. Br. J. Cancer.

[24] Corn K.C., Windham M.A., Rafat M. (2020). Lipids in the tumor microenvironment: From cancer progression to treatment. Prog. Lipid Res..

[25] Liao Z., Chua D., Tan N.S. (2019). Reactive oxygen species: A volatile driver of field cancerization and metastasis. Mol. Cancer.

[26] Yin H., Gu P., Xie Y., You X., Zhang Y., Yao Y., Yang S., Wang D., Chen W., Ma J. (2024). ALKBH5 mediates silica particles-induced pulmonary inflammation through increased m6A modification of Slamf7 and autophagy dysfunction. J. Hazard. Mater..

[27] Jere S.W., Houreld N.N., Abrahamse H. (2019). Role of the PI3K/AKT (mTOR and GSK3β) signalling pathway and photobiomodulation in diabetic wound healing. Cytokine Growth Factor. Rev..

[28] Taniguchi K., Karin M. (2018). NF-κB, inflammation, immunity and cancer: Coming of age. Nat. Rev. Immunol..

[29] Iqbal M.J., Kabeer A., Abbas Z., Siddiqui H.A., Calina D., Sharifi-Rad J., Cho W.C. (2024). Interplay of oxidative stress, cellular communication and signaling pathways in cancer. Cell Commun. Signal..

[30] Hayes J.D., Dinkova-Kostova A.T., Tew K.D. (2020). Oxidative stress in cancer. Cancer Cell.

[31] Sevcikova A., Izoldova N., Stevurkova V., Kasperova B., Chovanec M., Ciernikova S., Mego M. (2022). The Impact of the Microbiome on Resistance to Cancer Treatment with Chemotherapeutic Agents and Immunotherapy. Int. J. Mol. Sci..

[32] Wu N., Feng Y.Q., Lyu N., Wang D., Yu W.D., Hu Y.F. (2022). Fusobacterium nucleatum promotes colon cancer progression by changing the mucosal microbiota and colon transcriptome in a mouse model. World J. Gastroenterol..

[33] Trefny M.P., Kroemer G., Zitvogel L., Kobold S. (2025). Metabolites as agents and targets for cancer immunotherapy. Nat. Rev. Drug Discov..

[34] Zhai Z., Li X., Shang S., Ma S., Liang X., Yin S., Wu M., Yu J., Song Q., Chen D. (2026). Intratumoral microbiota and metabolites: Dual roles in cancer progression and therapeutic opportunities. Cell Commun. Signal..

[35] Gao W., Liu Y.F., Zhang Y.X., Wang Y., Jin Y.-Q., Yuan H., Liang X.-Y., Ji X.-Y., Jiang Q.-Y., Wu D.-D. (2024). The potential role of hydrogen sulfide in cancer cell apoptosis. Cell Death Discov..

[36] Lehouritis P., Springer C., Tangney M. (2013). Bacterial-directed enzyme prodrug therapy. J. Control Release.

[37] Yang Q., Qin B., Hou W., Qin H., Yin F. (2023). Pathogenesis and therapy of radiation enteritis with gut microbiota. Front. Pharmacol..

[38] Azzimonti B., Ballacchino C., Zanetta P., Cucci M.A., Monge C., Grattarola M., Dianzani C., Barrera G., Pizzimenti S. (2023). Microbiota, Oxidative Stress, and Skin Cancer: An Unexpected Triangle. Antioxidants.

[39] Perillo B., Di Donato M., Pezone A., Perillo B., Di Donato M., Pezone A., Di Zazzo E., Giovannelli P., Galasso G., Castoria G. (2020). ROS in cancer therapy: The bright side of the moon. Exp. Mol. Med..

[40] An X., Yu W., Liu J., An X., Yu W., Liu J., Tang D., Yang L., Chen X. (2024). Oxidative cell death in cancer: Mechanisms and therapeutic opportunities. Cell Death Dis..

[41] Piskounova E., Agathocleous M., Murphy M.M., Piskounova E., Agathocleous M., Murphy M.M., Hu Z., Huddlestun S.E., Zhao Z., Leitch A.M. (2015). Oxidative stress inhibits distant metastasis by human melanoma cells. Nature.

[42] Song Y., Hou Z., Zhu L., Chen Y., Li J. (2025). Oxidative stress as a catalyst in prostate cancer progression: Unraveling molecular mechanisms and exploring therapeutic interventions. Discov. Oncol..

[43] Wei H., Xiong M., Min L. (2025). ROS-mediated cell death and phase separation in gynecological malignancies. Eur. J. Med. Res..

[44] Wang Y., Branicky R., Noë A., Hekimi S. (2018). Superoxide dismutases: Dual roles in controlling ROS damage and regulating ROS signaling. J. Cell. Physiol..

[45] Sepasi Tehrani H., Moosavi-Movahedi A.A. (2018). Catalase and its mysteries. Prog. Biophys. Mol. Biol..

[46] Handy D.E., Loscalzo J. (2022). The role of glutathione peroxidase-1 in health and disease. Free Radic. Biol. Med..

[47] Bortolotti M., Polito L., Battelli M.G., Bolognes I.A. (2021). Xanthine oxidoreductase: One enzyme, multiple physiological and pathological roles. Int. J. Mol. Sci..

[48] Arnandis T., Monteiro P., Adams S.D., Bridgeman V.L., Rajeeve V., Gadaleta E., Marzec J., Chelala C., Malanchi I., Cutillas P.R. (2018). Oxidative stress in cells with extra centrosomes drives non-cell-autonomous invasion. Dev. Cell.

[49] Igelmann S., Neubauer H.A., Ferbeyre G. (2019). STAT3 and STAT5 activation in solid cancers. Cancers.

[50] Harris I.S., Treloar A.E., Inoue S., Sasaki M., Gorrini C., Lee K.C., Yung K.Y., Brenner D., Knobbe-Thomsen C.B., Cox M.A. (2015). Glutathione and thioredoxin antioxidant pathways synergize to drive cancer initiation and progression. Cancer Cell.

[51] Koch A., Ebert E.V., Seitz T., Dietrich P., Berneburg M., Bosserhof A., Hellerbrand C. (2020). Characterization of glycolysis-related gene expression in malignant melanoma. Pathol. Res. Pract..

[52] Franklin C.C., Backos D.S., Mohar I., White C.C., Forman H.J., Kavanagh T.J. (2009). Structure, function, and post-translational regulation of the catalytic and modifier subunits of glutamate cysteine ligase. Mol. Asp. Med..

[53] Ramos-Gomez M., Kwak M.K., Dolan P.M., Itoh K., Yamamoto M., Talalay P., Kensler T.W. (2001). Sensitivity to carcinogenesis is increased and chemoprotective efficacy of enzyme inducers is lost in nrf2 transcription factor-deficient mice. Proc. Natl. Acad. Sci. USA.

[54] Romero R., Sayin V.I., Davidson S.M., Bauer M.R., Singh S.X., LeBoeuf S.E., Karakousi T.R., Ellis D.C., Bhutkar A., Sánchez-Rivera F.J. (2017). Keap1 loss promotes Kras-driven lung cancer and results in dependence on glutaminolysis. Nat. Med..

[55] Son J., Lyssiotis C.A., Ying H., Wang X., Hua S., Ligorio M., Perera R.M., Ferrone C.R., Mullarky E., Shyh-Chang N. (2013). Glutamine supports pancreatic cancer growth through a KRAS-regulated metabolic pathway. Nature.

[56] Ge T., Yang J., Zhou S., Wang Y., Li Y., Tong X. (2020). The role of the pentose phosphate pathway in cancer. Front. Oncol..

[57] Ying H., Kimmelman A.C., Lyssiotis C.A., Hua S., Chu G.C., Fletcher-Sananikone E., Locasale J.W., Son J., Zhang H., Coloff J.L. (2012). Oncogenic Kras maintains pancreatic tumors through regulation of anabolic glucose metabolism. Cell.

[58] Kim S.Y., Adhikari A., Lee S.Y., Marshel J.H., Kim C.K., Mallory C.S., Lo M., Pak S., Mattis J., Lim B.K. (2013). Diverging neural pathways assemble a behavioural state from separable features in anxiety. Nature.

[59] Mitsuishi Y., Taguchi K., Kawatani Y., Shibata T., Nukiwa T., Aburatani H., Yamamoto M., Motohashi H. (2012). Nrf2 redirects glucose and glutamine into anabolic pathways in metabolic reprogramming. Cancer Cell.

[60] Jiang P., Du W., Wang X., Mancuso A., Gao X., Wu M., Yang X. (2011). p53 regulates biosynthesis through direct inactivation of glucose-6-phosphate dehydrogenase. Nat. Cell Biol..

[61] Nakamura M., Magara T., Yoshimitsu M., Kano S., Kato H., Yokota K., Okuda K., Morita A. (2024). Blockade of glucose-6-phosphate dehydrogenase induces immunogenic cell death and accelerates immunotherapy. J. Immunother. Cancer.

[62] Bensaad K., Tsuruta A., Selak M.A., Vidal M.N., Nakano K., Bartrons R., Gottlieb E., Vousden K.H. (2006). TIGAR, a p53-inducible regulator of glycolysis and apoptosis. Cell.

[63] Kang M.Y., Kim H.B., Piao C., Lee K.H., Hyun J.W., Chang I.Y., You H.J. (2013). The critical role of catalase in prooxidant and antioxidant function of p53. Cell Death Differ..

[64] Jiang L., Kon N., Li T., Wang S.J., Su T., Hibshoosh H., Baer R., Gu W. (2015). Ferroptosis as a p53-mediated activity during tumour suppression. Nature.

[65] Huo Y., Yin S., Yan M., Win S. (2017). Protective role of p53 in acetaminophen hepatotoxicity. Free Radic. Biol. Med..

[66] Humpton T.J., Vousden K.H. (2016). Regulation of cellular metabolism and hypoxia by p53. Cold Spring Harb. Perspect. Med..

[67] Humpton T.J., Hock A.K., Maddocks O.D.K., Vousden K.H. (2018). P53-mediated adaptation to serine starvation is retained by a common tumour-derived mutant. Cancer Metab..

[68] Aanniz T., El Fessikh M., Touhtouh J., Aboulaghras S., El Omari N., Khalid A., Abdalla A.N., Amanullah M., Goh B.H., Lee L.H. (2025). TET enzymes: Involvement in cancer development and therapeutical perspectives. Biochim. Biophys. Acta Gene Regul. Mech..

[69] Nishiyama A., Nakanishi M. (2021). Navigating the DNA methylation landscape of cancer. Trends Genet..

[70] García-Giménez J.L., Garcés C., Romá-Mateo C., Pallardó F.V. (2021). Oxidative stress-mediated alterations in histone post-translational modifications. Free Radic. Biol. Med..

[71] Park M.N. (2026). Redox-Guided Epigenetic Signaling in Cancer: miRNA–DNMT Feedback Loops as Epigenetic Memory Modulates. Antioxidants.

[72] Zhu W.-G. (2020). The roles of histone deacetylases and their inhibitors in cancer therapy. Front. Cell Dev. Biol..

[73] Daniel F.I., Cherubini K., Yurgel L.S., de Figueiredo M.A., Salum F.G. (2011). The role of epigenetic transcription repression and DNA methyltransferases in cancer. Cancer.

[74] Balamurli G., Liew A.Q.X., Tee W.W., Pervaiz S. (2024). Interplay between epigenetics, senescence and cellular redox metabolism in cancer and its therapeutic implications. Redox Biol..

[75] Zuo J., Zhang Z., Li M., Yang Y., Zheng B., Wang P., Huang C., Zhou S. (2022). The crosstalk between reactive oxygen species and noncoding RNAs: From cancer code to drug role. Mol. Cancer.

[76] Mateescu B., Batista L., Cardon M., Gruosso T., de Feraudy Y., Mariani O., Nicolas A., Meyniel J.P., Cottu P., Sastre-Garau X. (2011). MiR-141 and miR-200a act on ovarian tumorigenesis by controlling oxidative stress response. Nat. Med..

[77] Liu C., Su C., Chen Y., Li G. (2018). MiR-144-3p promotes the tumor growth and metastasis of papillary thyroid carcinoma by targeting paired box gene 8. Cancer Cell Int..

[78] Fu J., Imani S., Wu M.Y., Wu R.C. (2023). MicroRNA-34 Family in Cancers: Role, Mechanism, and Therapeutic Potential. Cancers.

[79] Dang K., Myers K.A. (2015). The role of hypoxia-induced miR-210 in cancer progression. Int. J. Mol. Sci..

[80] Fabrizio F.P., Sparaneo A., Muscarella L.A. (2020). NRF2 Regulation by Noncoding RNAs in Cancers: The Present Knowledge and the Way Forward. Cancers.

[81] Wu X.S., Wang X.A., Wu W.G., Hu Y.P., Li M.L., Ding Q., Weng H., Shu Y.J., Liu T.Y., Jiang L. (2014). MALAT1 promotes the proliferation and metastasis of gallbladder cancer cells by activating the ERK/MAPK pathway. Cancer Biol. Ther..

[82] Wu W., Yao Z., Chen Y., Xu R., Jin C., Li X. (2025). HOTAIR, a ferroptosis-related gene, promotes malignant behavior of breast cancer via sponging miR-206. Discov. Oncol..

[83] Baba S.K., Baba S.K., Mir R., Elfaki I., Algehainy N., Ullah M.F., Barnawi J., Altemani F.H., Alanazi M., Mustafa S.K. (2023). Long non-coding RNAs modulate tumor microenvironment to promote metastasis: Novel avenue for therapeutic intervention. Front. Cell Dev. Biol..

[84] Roso-Mares A., Andújar I., Díaz Corpas T., Sun B.K. (2024). Non-coding RNAs as skin disease biomarkers, molecular signatures, and therapeutic targets. Hum. Genet..

[85] Gorgoulis V., Adams P.D., Alimonti A., Bennett D.C., Bischof O., Bishop C., Campisi J., Collado M., Evangelou K., Ferbeyre G. (2019). Cellular Senescence: Defining a Path Forward. Cell.

[86] Nadeem J., Sultana R., Parveen A., Kim S.Y. (2025). Recent Advances in Anti-Aging Therapeutic Strategies Targeting DNA Damage Response and Senescence-Associated Secretory Phenotype-Linked Signaling Cascade. Cell Biochem. Funct..

[87] Ozsarlak-Sozer G., Kerry Z., Gokce G., Oran I., Topcu Z. (2011). Oxidative stress in relation to telomere length maintenance in vascular smooth muscle cells following balloon angioplasty. J. Physiol. Biochem..

[88] Collado M., Serrano M. (2022). The senescence-associated secretory phenotype: A new frontier in cancer. Nat. Rev. Cancer.

[89] Malavolta M., Bracci M., Santarelli L., Sayeed M.A., Pierpaoli E., Giacconi R., Costarelli L., Piacenza F., Basso A., Cardelli M. (2018). Inducers of Senescence, Toxic Compounds, and Senolytics: The Multiple Faces of Nrf2-Activating Phytochemicals in Cancer Adjuvant Therapy. Mediat. Inflamm..

[90] Ewald J.A., Desotelle J.A., Wilding G., Jarrard D.F. (2010). Therapy-induced senescence in cancer. J. Natl. Cancer Inst..

[91] Birch J., Gil J. (2020). Senescence and the SASP: Many therapeutic avenues. Genes Dev..

[92] Jin P., Feng X.-D., Huang C.-S., Li J., Wang H., Wang X.-M., Li L., Ma L.-Q. (2024). Oxidative stress and cellular senescence: Roles in tumor progression and therapeutic opportunities. MedComm–Oncol..

[93] Kirkland J.L., Tchkonia T. (2020). Senolytic drugs: From discovery to translation. J. Intern. Med..

[94] He X.-Y., Gao Y., Ng D., Michalopoulou E., George S., Adrover J.M., Sun L., Albrengues J., Daßler-Plenker J., Han X. (2024). Chronic stress increases metastasis via neutrophil-mediated changes to the microenvironment. Cancer Cell.

[95] Xu H., Hu C., Wang Y., Shi Y., Yuan L., Xu J., Zhang Y., Chen J., Wei Q., Qin J. (2023). Glutathione peroxidase 2 knockdown suppresses gastric cancer progression and metastasis via regulation of kynurenine metabolism. Oncogene.

[96] Sadik A., Somarribas Patterson L.F., Öztürk S., Mohapatra S.R., Panitz V., Secker P.F., Pfänder P., Loth S., Salem H., Prentzell M.T. (2020). IL4I1 Is a Metabolic Immune Checkpoint that Activates the AHR and Promotes Tumor Progression. Cell.

[97] Liu Y., Niu R., Deng R., Wang Y., Song S., Zhang H. (2024). Multi-Enzyme Co-Expressed Nanomedicine for Anti-Metastasis Tumor Therapy by Up-Regulating Cellular Oxidative Stress and Depleting Cholesterol. Adv. Mater..

[98] Torti S.V., Torti F.M. (2013). Iron and cancer: More ore to be mined. Nat. Rev. Cancer.

[99] Chen S., Yu C., Kang R., Tang D. (2020). Iron metabolism in ferroptosis. Front. Cell Dev. Biol..

[100] Chen L., Min J., Wang F. (2022). Copper homeostasis and cuproptosis in health and disease. Signal Transduct. Target. Ther..

[101] Bejarano L., Jordāo M.J.C., Joyce J.A. (2021). Therapeutic targeting of the tumor microenvironment. Cancer Discov..

[102] Aboelella N.S., Brandle C., Kim T., Ding Z.-C., Zhou G. (2021). Oxidative stress in the tumor microenvironment and its relevance to cancer immunotherapy. Cancers.

[103] Zhang M., Guo X., Wang M., Liu K. (2020). Tumor microenvironment-induced structure changing drug/gene delivery system for overcoming delivery-associated challenges. J. Control. Release.

[104] Babior B.M., Kipnes R.S., Curnutte J.T. (1973). Biological defense mechanisms. The production by leukocytes of superoxide, a potential bactericidal agent. J. Clin. Investig..

[105] Jackson S.H., Devadas S., Kwon J., Pinto L.A., Williams M.S. (2004). T cells express a phagocyte-type NADPH oxidase that is activated after T cell receptor stimulation. Nat. Immunol..

[106] Kesarwani P., Murali A.K., Al-Khami A.A., Mehrotra S. (2013). Redox regulation of T-cell function: From molecular mechanisms to significance in human health and disease. Antioxid. Redox Signal..

[107] Yang P.-X., Fan X.-X., Liu M.-X., Zhang X.-Z., Cao L., Wang Z.-Z., Tian J.-Z., Zhang Y.-W., Xiao W. (2024). Longxuetongluo capsule alleviate ischemia/reperfusion induced cardiomyocyte apoptosis through modulating oxidative stress and mitochondrial dysfunction. Phytomedicine.

[108] Corazzari M., Piacentini M. (2017). Endoplasmic reticulum stress, unfolded protein response, and cancer cell fate. Front. Oncol..

[109] Liberti M.V., Locasale J.W. (2016). The Warburg Effect: How Does it Benefit Cancer Cells?. Trends Biochem. Sci..

[110] Battello N., Zimmer A.D., Goebel C., Dong X., Behrmann I., Haan C., Hiller K., Wegner A. (2016). The role of HIF-1 in oncostatin M-dependent metabolic reprogramming of hepatic cells. Cancer Metab..

[111] Nathan C., Cunningham-Bussel A. (2013). Beyond oxidative stress: An immunologist’s guide to reactive oxygen species. Nat. Rev. Immunol..

[112] Wang B., Wang Y., Zhang J., Hu C., Jiang J., Li Y., Peng Z. (2023). ROS-induced lipid peroxidation modulates cell death outcome: Mechanisms behind apoptosis, autophagy, and ferroptosis. Arch. Toxicol..

[113] Wang Y., Huang J., Tong H., Jiang Y., Jiang Y., Ma X. (2025). Nutrient acquisition of gut microbiota: Implications for tumor immunity. Semin. Cancer Biol..

[114] Scharping N.E., Rivadeneira D.B., Menk A.V., Vignali P.D.A., Ford B.R., Rittenhouse N.L., Peralta R., Wang Y., Wang Y., DePeaux K. (2021). Mitochondrial stress induced by continuous stimulation under hypoxia rapidly drives T cell exhaustion. Nat. Immunol..

[115] Wu Z., Zuo M., Zeng L., Cui K., Liu B., Yan C., Chen L., Dong J., Shangguan F., Hu W. (2021). OMA1 reprograms metabolism under hypoxia to promote colorectal cancer development. EMBO Rep..

[116] Lopez Krol A., Nehring H.P., Krause F.F., Wempe A., Raifer H., Nist A., Stiewe T., Bertrams W., Schmeck B., Luu M. (2022). Lactate induces metabolic and epigenetic reprogramming of pro-inflammatory Th17 cells. EMBO Rep..

[117] Balta E., Janzen N., Kirchgessner H., Toufaki V., Orlik C., Liang J., Lairikyengbam D., Abken H., Niesler B., Müller-Decker K. (2022). Expression of TRX1 optimizes the antitumor functions of human CAR T cells and confers resistance to a pro-oxidative tumor microenvironment. Front. Immunol..

[118] Chu Y., Lan R.S., Huang R., Feng H., Kumar R., Dayal S., Chan K., Dai D. (2020). Glutathione peroxidase-1 overexpression reduces oxidative stress, and improves pathology and proteome remodeling in the kidneys of old mice. Aging Cell.

[119] Abu Shelbayeh O., Arroum T., Morris S., Busch K.B. (2023). PGC-1α is a master regulator of mitochondrial lifecycle and ROS stress response. Antioxidants.

[120] Zhong X., Wu H., Ouyang C., Zhang W., Shi Y., Wang Y.-C., Ann D.K., Gwack Y., Shang W., Sun Z. (2023). Ncoa2 promotes CD8+ T cell-mediated antitumor immunity by stimulating T-cell activation via upregulation of PGC-1α critical for mitochondrial function. Cancer Immunol. Res..

[121] Lontos K., Wang Y., Joshi S.K., Frisch A.T., Watson M.J., Kumar A., Menk A.V., Wang Y., Cumberland R., Lohmueller J. (2023). Metabolic reprogramming via an engineered PGC-1α improves human chimeric antigen receptor T-cell therapy against solid tumors. J. Immunother. Cancer.

[122] Renken S., Nakajima T., Magalhaes I., Mattsson J., Lundqvist A., Arnér E.S.J., Kiessling R., Wickström S.L. (2022). Targeting of Nrf2 improves antitumoral responses by human NK cells, TIL and CAR T cells during oxidative stress. J. Immunother. Cancer.

[123] Zhang Y., Tian S., Huang L., Li Y., Lu Y., Li H., Chen G., Meng F., Liu G.L., Yang X. (2022). Reactive oxygen species-responsive and Raman-traceable hydrogel combining photodynamic and immune therapy for postsurgical cancer treatment. Nat. Commun..

[124] Lu S.-P., Lin Feng M.-H., Huang H.-L., Huang Y.-C., Tsou W.-I., Lai M.-Z. (2007). Reactive oxygen species promote raft formation in T lymphocytes. Free Radic. Biol. Med..

[125] Gao H., Nepovimova E., Heger Z., Valko M., Wu Q., Kuca K., Adam V. (2023). Role of hypoxia in cellular senescence. Pharmacol. Res..

[126] Ichijo H., Nishida E., Irie K., Ten Dijke P., Saitoh M., Moriguchi T., Takagi M., Matsumoto K., Miyazono K., Gotoh Y. (1997). Induction of apoptosis by ASK1, a mammalian MAPKKK that activates SAPK/JNK and p38 signaling pathways. Science.

[127] Tobiume K., Matsuzawa A., Takahashi T., Nishitoh H., Morita K., Takeda K., Minowa O., Miyazono K., Noda T., Ichijo H. (2001). ASK1 is required for sustained activations of JNK/p38 MAP kinases and apoptosis. EMBO Rep..

[128] Han J., Sun P. (2007). The pathways to tumor suppression via route p38. Trends Biochem. Sci..

[129] Nakamura M., Ohsawa S., Igaki T. (2014). Mitochondrial defects trigger proliferation of neighbouring cells via a senescence-associated secretory phenotype in Drosophila. Nat. Commun..

[130] Dixon S.J., Olzmann J.A. (2024). The cell biology of ferroptosis. Nat. Rev. Mol. Cell Biol..

[131] Fan X., Li A., Yan Z., Geng X., Lian L., Lv H., Gao D., Zhang J. (2022). From iron metabolism to ferroptosis: Pathologic changes in coronary heart disease. Oxidative Med. Cell. Longev..

[132] Zhou Q., Meng Y., Li D., Yao L., Le J., Liu Y., Sun Y., Zeng F., Chen X., Deng G. (2024). Ferroptosis in cancer: From molecular mechanisms to therapeutic strategies. Signal Transduct. Target. Ther..

[133] Yang W.S., SriRamaratnam R., Welsch M.E., Shimada K., Skouta R., Viswanathan V.S., Cheah J.H., Clemons P.A., Shamji A.F., Clish C.B. (2014). Regulation of ferroptotic cancer cell death by GPX4. Cell.

[134] Stockwell B.R., Friedmann Angeli J.P., Bayir H., Bush A.I., Conrad M., Dixon S.J., Fulda S., Gascón S., Hatzios S.K., Kagan V.E. (2017). Ferroptosis: A regulated cell death nexus linking metabolism, redox biology, and disease. Cell.

[135] Jiang X., Stockwell B.R., Conrad M. (2021). Ferroptosis: Mechanisms, biology and role in disease. Nat. Rev. Mol. Cell Biol..

[136] Thamilselvan V., Menon M., Stein G.S., Valeriote F., Thamilselvan S. (2017). Combination of carmustine and selenite inhibits EGFR mediated growth signaling in androgen-independent prostate cancer cells. J. Cell. Biochem..

[137] Ewend M.G., Brem S., Gilbert M., Goodkin R., Penar P.L., Varia M., Cush S., Carey L.A. (2007). Treatment of single brain metastasis with resection, intracavity carmustine polymer wafers, and radiation therapy is safe and provides excellent local control. Clin. Cancer Res..

[138] Huang R., Chen H., Liang J., Li Y., Yang J., Luo C., Tang Y., Ding Y., Liu X., Yuan Q. (2021). Dual role of reactive oxygen species and their application in cancer therapy. J. Cancer.

[139] Pei X., Xiao J., Wei G., Zhang Y. (2019). Oenothein B inhibits human non-small cell lung cancer A549 cell proliferation by ROS-mediated PI3K/Akt/NF-κB signaling pathway. Chem.-Biol. Interact..

[140] Su X., Shen Z., Yang Q., Sui F., Pu J., Ma J., Ma S., Yao D., Ji M., Hou P. (2019). Vitamin C kills thyroid cancer cells through ROS-dependent inhibition of MAPK/ERK and PI3K/AKT pathways via distinct mechanisms. Theranostics.

[141] Yuan Z., Liang Z., Yi J., Chen X., Li R., Wu J., Sun Z. (2019). Koumine promotes ROS production to suppress hepatocellular carcinoma cell proliferation via NF-κB and ERK/p38 MAPK signaling. Biomolecules.

[142] Li Y., Liang R., Zhang X., Wang J. (2019). Copper chaperone for superoxide dismutase promotes breast cancer cell proliferation and migration via ROS-mediated MAPK/ERK signaling. Front. Pharmacol..

[143] Redza-Dutordoir M., Averill-Bates D.A. (2016). Activation of apoptosis signalling pathways by reactive oxygen species. Biochim. Biophys. Acta.

[144] Fleury C., Mignotte B., Vayssière J.L. (2002). Mitochondrial reactive oxygen species in cell death signaling. Biochimie.

[145] Peña-Blanco A., García-Sáez A.J. (2018). Bax, Bak and beyond—Mitochondrial performance in apoptosis. FEBS J..

[146] Adamska A., Stefanowicz-Hajduk J., Ochocka J.R. (2019). Alpha-hederin, the active saponin of *Nigella sativa*, as an anticancer agent inducing apoptosis in the SKOV-3 cell line. Molecules.

[147] Wang J., Deng H., Zhang J., Wu D., Li J., Ma J., Dong W. (2020). α-Hederin induces the apoptosis of gastric cancer cells accompanied by glutathione decrement and reactive oxygen species generation via activating mitochondrial dependent pathway. Phytother. Res..

[148] Yu B., Liu Y., Peng X., Hua S., Zhou G., Yan K., Liu Y. (2020). Synthesis, characterization, and antitumor properties of Au(I)-thiourea complexes. Metallomics.

[149] Hernández Borrero L.J., El-Deiry W.S. (2021). Tumor suppressor p53: Biology, signaling pathways, and therapeutic targeting. Biochim. Biophys. Acta Rev. Cancer.

[150] Aubrey B.J., Kelly G.L., Janic A., Herold M.J., Strasser A. (2018). How does p53 induce apoptosis and how does this relate to p53-mediated tumour suppression?. Cell Death Differ..

[151] Shi T., Dansen T.B. (2020). Reactive oxygen species induced p53 activation: DNA damage, redox signaling, or both?. Antioxid. Redox Signal..

[152] Punganuru S.R., Madala H.R., Arutla V., Srivenugopal K.S. (2018). Selective killing of human breast cancer cells by the styryl lactone (R)-goniothalamin is mediated by glutathione conjugation, induction of oxidative stress and marked reactivation of the R175H mutant p53 protein. Carcinogenesis.

[153] Basak D., Punganuru S.R., Srivenugopal K.S. (2016). Piperlongumine exerts cytotoxic effects against cancer cells with mutant p53 proteins at least in part by restoring the biological functions of the tumor suppressor. Int. J. Oncol..

[154] Valko M., Jomova K., Rhodes C.J., Kuča K., Musílek K. (2016). Redox- and non-redox-metal-induced formation of free radicals and their role in human disease. Arch. Toxicol..

[155] Shao J., Li M., Guo Z., Jin C., Zhang F., Ou C., Xie Y., Tan S., Wang Z., Zheng S. (2019). TPP-related mitochondrial targeting copper (II) complex induces p53-dependent apoptosis in hepatoma cells through ROS-mediated activation of Drp1. Cell Commun. Signal..

[156] Ahmad Bhat I., Maqsood Bhat A., Tasduq Abdullah S. (2025). Apoptosis mechanisms, regulation in pathology, and therapeutic potential. Cell Death Regulation in Pathology.

[157] Tsai C.C., Wang C.Y., Chang H.H., Chang P.T.S., Chang C.H., Chu T.Y., Hsu P.C., Kuo C.Y. (2024). Diagnostics and therapy for malignant tumors. Biomedicines.

[158] Kisby T., Borst G.R., Coope D.J., Kostarelos K. (2025). Targeting the glioblastoma resection margin with locoregional nanotechnologies. Nat. Rev. Clin. Oncol..

[159] Zhou Z., Pang Y., Ji J., He J., Liu T., Ouyang L., Zhang W., Zhang X.L., Zhang Z.G., Zhang K. (2023). Harnessing 3D in vitro systems to model immune responses to solid tumours: A step towards improving and creating personalized immunotherapies. Nat. Rev. Immunol..

[160] Arfin S., Jha N.K., Jha S.K., Kesari K.K., Ruokolainen J., Roychoudhury S., Rathi B., Kumar D. (2021). Oxidative stress in cancer cell metabolism. Antioxidants.

[161] Liu Y., Liu Y.Q., Zang J., Abdullah A.A.I., Li Y.Y., Dong H.Q. (2020). Design strategies and applications of ROS-responsive phenylborate ester-based nanomedicine. ACS Biomater. Sci. Eng..

[162] Song C.C., Ji R., Du F.S., Liang D.H., Li Z.C. (2013). Oxidation-accelerated hydrolysis of the ortho ester-containing acid-labile polymers. ACS Macro Lett..

[163] Rinaldi A., Caraffi R., Grazioli M.V., Oddone N., Giardino L., Tosi G., Vandelli M.A., Calzà L., Ruozi B., Duskey J.T. (2022). Applications of the ROS-responsive thioketal linker for the production of smart nanomedicines. Polymers.

[164] Liu B., Thayumanavan S. (2020). Mechanistic investigation on oxidative degradation of ROS-responsive thioacetal/thioketal moieties and their implications. Cell Rep. Phys. Sci..

[165] Hu J.C., Zhang Q.H., Mu Q.Q., Tang Y.Y., Wu Z., Wang G.J. (2022). A ROS-sensitive diselenide-crosslinked polymeric nanogel for NIR controlled release. Chin. J. Polym. Sci..

[166] Bio M., Nkepang G., You Y. (2012). Click and photo-unclick chemistry of aminoacrylate for visible light-triggered drug release. Chem. Commun..

[167] Chi T., Sang T., Wang Y., Ye Z. (2023). Cleavage and noncleavage chemistry in reactive oxygen species (ROS)-responsive materials for smart drug delivery. Bioconjug. Chem..

[168] Shim M.S., Xia Y. (2013). A reactive oxygen species (ROS)-responsive polymer for safe, efficient, and targeted gene delivery in cancer cells. Angew. Chem. Int. Ed..

[169] Xiao C., Ding J., Ma L., Yang C., Zhuang X., Chen X. (2015). Synthesis of thermal and oxidation dual responsive polymers for reactive oxygen species (ROS)-triggered drug release. Polym. Chem..

[170] Kim D.H., Rozhkova E.A., Ulasov I.V., Bader S.D., Rajh T., Lesniak M.S., Novosad V. (2010). Biofunctionalized magnetic-vortex microdiscs for targeted cancer-cell destruction. Nat. Mater..

[171] Shen S., Yan Z., Wu J., Liu X., Guan G., Zou C., Guo Q., Zhu C., Liu T., Chen C. (2020). Characterization of ROS metabolic equilibrium reclassifies pan-cancer samples and guides pathway targeting therapy. Front. Oncol..

[172] Ji X.Y., Pan Z.X., Yu B.C., De la Cruz L.K., Zheng Y.Q., Ke B.W., Wang B. (2019). Click and release: Bioorthogonal approaches to “on-demand” activation of prodrugs. Chem. Soc. Rev..

[173] Hu Q.W., Yammani R.D., Brown-Harding H., Soto-Pantoja D.R., Poole L.B., Lukesh J. (2022). Mitigation of doxorubicin-induced cardiotoxicity with an H_2_O_2_-activated, H_2_S-donating hybrid prodrug. Redox Biol..

[174] Bielec B., Poetsch I., Ahmed E., Heffeter P., Keppler B.K., Kowol C.R. (2020). Reactive oxygen species (ROS)-sensitive prodrugs of the tyrosine kinase inhibitor crizotinib. Molecules.

[175] Zhu J.W., Chen J.T., Song D.M., Zhang W.D., Guo J.P., Cai G.P., Ren Y., Wan C., Kong L., Yu W. (2019). Real-time monitoring of etoposide prodrug activated by hydrogen peroxide with improved safety. J. Mater. Chem. B.

[176] Gong Q.J., Li X., Li T., Wu X.S., Hu J.B., Yang F.L., Zhang X. (2022). A carbon-carbon bond cleavage-based prodrug activation strategy applied to β-Lapachone for cancer-specific targeting. Angew. Chem. Int. Ed..

[177] Xu Z., Wang H., Liu H., Chen H., Jiang B. (2022). Synthesis and evaluation of reactive oxygen species sensitive prodrugs of a NAMPT inhibitor FK866. Molecules.

[178] Reshetnikov V., Özkan H.G., Daum S., Janko C., Alexiou C., Sauer C., Heinrich M.R., Mokhir A. (2020). N-alkylaminoferrocene-based prodrugs targeting mitochondria of cancer cells. Molecules.

[179] Xu H.G., Annamadov S., Mokhir A. (2022). 4-Ferrocenylaniline-based ROS-responsive prodrugs with anticancer activity. J. Organomet. Chem..

[180] Zhang Q.E., Fan X.J., Qian H.M., Xiao S.S., Song Q., Wang Y.C., Wang J., Yang S., Wang Y. (2025). Synthesis and bio-evaluation of aminoferrocene-based anticancer prodrugs as potent ferroptosis inducers. Inorg. Chem. Front..

[181] Yang C.Y., Yu P.Y., Chen J.X., Lu R.X., Hai L., Yang Z.Z., Guo L., Wu Y. (2025). An oxidation-reduction-triggered thiamine disulfide-based prodrug of 10-hydroxycamptothecin for selective tumor cell locking and therapeutic delivery. Eur. J. Med. Chem..

[182] He Z.L., Charleton C., Devine R.W., Kelada M., Walsh J.M.D., Conway G.E., Gunes S., Mondala J.R.M., Tian F., Tiwari B. (2021). Enhanced pyrazolopyrimidinones cytotoxicity against glioblastoma cells activated by ROS-generating cold atmospheric plasma. Eur. J. Med. Chem..

[183] Luo X.J., Chi X.Q., Lin Y.Y., Yang Z.X., Lin H.Y., Gao J.H. (2021). A camptothecin prodrug induces mitochondria-mediated apoptosis in cancer cells with cascade activations. Chem. Commun..

[184] Thapa P., Li M.J., Bio M., Rajaputra P., Nkepang G., Sun Y.J., Woo S., You Y. (2016). Far-red light-activatable prodrug of paclitaxel for the combined effects of photodynamic therapy and site-specific paclitaxel chemotherapy. J. Med. Chem..

[185] Fu Q., Li H., Duan D., Wang C., Shen S., Ma H., Liu Z. (2020). External-radiation-induced local hydroxylation enables remote release of functional molecules in tumors. Angew. Chem. Int. Ed..

[186] Wei D.S., Sun Y., Zhu H., Fu Q.R. (2023). Stimuli-responsive polymer-based nanosystems for cancer theranostics. ACS Nano.

[187] Gao C.X., Wang X.J., Yang B., Yuan W., Huang W., Wu G.J., Ma J. (2023). Synergistic target of intratumoral microbiome and tumor by metronidazole-fluorouridine nanoparticles. ACS Nano.

[188] Gong Y.H., Shu M., Xie J.H., Zhang C., Cao Z., Jiang Z.Z., Liu J. (2019). Enzymatic synthesis of PEG-poly(amine-thioether esters) as highly efficient pH and ROS dual-responsive nanocarriers for anticancer drug delivery. J. Mater. Chem. B.

[189] Yu L., Ke H.L., Du F.S., Li Z.C. (2019). Redox-responsive fluorescent polycarbonates based on selenide for chemotherapy of triple-negative breast cancer. Biomacromolecules.

[190] Hu T., Liu L.W., Zhang C., Feng Q.Y., Wang Q.Y., Zhang J.L., Xu Z., Conghu L., Cheng X., Wu Y. (2023). Self-assembled α-tocopherol succinate dimer nanoparticles combining doxorubicin for increasing chemotherapy/oxidative therapy in 3D tumor spheroids. J. Drug Deliv. Sci. Technol..

[191] Pan Q.Q., Deng X., Gao W.X., Chang J., Pu Y.J., He B. (2020). ROS triggered cleavage of thioketal moiety to dissociate prodrug nanoparticles for chemotherapy. Colloids Surf. B Biointerfaces.

[192] Yin W., Ke W.D., Chen W.J., Xi L.C., Zhou Q.H., Mukerabigwi J.F., Ge Z. (2019). Integrated block copolymer prodrug nanoparticles for combination of tumor oxidative stress amplification and ROS-responsive drug release. Biomaterials.

[193] Wang B., Chen K., Zhang Q.F., Gu L., Luo Q., Li Z.Q., Gong Q., Zhang H., Gu Z., Luo K. (2021). ROS-responsive amphiphilic block copolymer-drug conjugate: Design, synthesis and potential as an efficient drug delivery system via a positive feedback strategy. Chem. Eng. J..

[194] Kim Y., Uthaman S., Pillarisetti S., Noh K., Huh K.M., Park I.K. (2020). Bioactivatable reactive oxygen species-sensitive nanoparticulate system for chemo-photodynamic therapy. Acta Biomater..

[195] He Y., Wang J., Wang S., Yu K., Zhou J., Wang J., Tang G., Gu Z., Bai H. (2023). Natural mussel protein-derived antitumor nanomedicine with tumor-targeted bioadhesion and penetration. Nano Today.

[196] Tan J.B., Jing P., Xiao X., Liao Y.L., Liao C.Y., Zhang S.Y. (2023). Cross-linked lipoic acid nanocapsules serve as H_2_O_2_ amplifier to strengthen the H_2_O_2_-sensitive prodrug activation. Sci. China Chem..

[197] Wang J., Zhang H.X., Lv J.Z., Zheng Y., Li M.Y., Yang G., Wei X., Li N., Huang H., Li T. (2023). A tumor-specific ROS self-supply enhanced cascade-responsive prodrug activation nanosystem for amplified chemotherapy against multidrug-resistant tumors. Acta Biomater..

[198] Xu X.Y., Zeng Z.S., Ding X., Shan T., Liu Q.X., Chen M.X., Chen J., Xia M., He Y., Huang Z. (2021). Reactive oxygen species-activatable self-amplifying Watson-Crick base pairing-inspired supramolecular nanoprodrug for tumor-specific therapy. Biomaterials.

[199] Hu Y.R., Zhao X.M., Liu P. (2024). Design of diselenide-containing polyprodrug and its pH/redox co-triggered degradation and slow drug release for tumor-specific chemotherapy. Colloids Surf. A Physicochem. Eng. Asp..

[200] Zheng Y.F., Qin C., Li F., Qi J.X., Chu X.Y., Li H., Shi T., Yan Z., Yang L., Xin X. (2023). Self-assembled thioether-bridged paclitaxel-dihydroartemisinin prodrug for amplified antitumor efficacy-based cancer ferroptotic-chemotherapy. Biomater. Sci..

[201] Zhang B.B., Liu H., Wang Y.F., Zhang Y. (2024). ROS-responsive and self-catalytic nanocarriers for a combination of chemotherapy and reinforced ferroptosis against breast cancer. ACS Biomater. Sci. Eng..

[202] Qin Y., Liu N., Wang F.H., Gao Z.P., Luo C., Tian C.T., Kamei K. (2025). Self-amplifying ROS-responsive SN38 prodrug nanoparticles for combined chemotherapy and ferroptosis in cancer treatment. Carbon.

[203] Yao X., Sun W., Yuan Y., Hu J., Fu J., Yin J. (2024). Amonafide-based H_2_O_2_-responsive theranostic prodrugs: Exploring the correlation between H_2_O_2_ level and anticancer efficacy. Bioorg Chem..

[204] Ahmadi M., Singer D., Potlitz F., Nasri Z., von Woedtke T., Link A., Bekeschus S., Wende K. (2023). Cold Physical Plasma-Mediated Fenretinide Prodrug Activation Confers Additive Cytotoxicity in Epithelial Cells. Antioxidants.

[205] Duan D., Dong H., Tu Z., Wang C., Fu Q., Chen J., Zhong H., Du P., Sun L.D., Liu Z. (2021). Desilylation Induced by Metal Fluoride Nanocrystals Enables Cleavage Chemistry In Vivo. J. Am. Chem. Soc..

[206] Zhang M., Asghar S., Tian C., Hu Z., Ping Q., Chen Z., Shao F., Xiao Y. (2021). Lactoferrin/phenylboronic acid-functionalized hyaluronic acid nanogels loading doxorubicin hydrochloride for targeting glioma. Carbohydr. Polym..

[207] Wang J., Yi J. (2008). Cancer cell killing via ROS: To increase or decrease, that is a question. Cancer Biol. Ther..

[208] Sies H., Jones D.P. (2021). The glutathione/glutathione disulfide ratio as a redox sensor. J. Biol. Chem..

[209] Eid M., Barayeu U., Dick T.P. (2026). Chemogenetic detection and quantitation of H_2_O_2_ in living cells. Nat. Protoc..

[210] Stockwell B.R. (2022). Ferroptosis turns 10: Emerging mechanisms, physiological functions, and therapeutic applications. Cell.

[211] Rochette L., Dogon G., Rigal E., Zeller M., Cottin Y., Vergely C. (2022). Lipid Peroxidation and Iron Metabolism: Two Corner Stones in the Homeostasis Control of Ferroptosis. Int. J. Mol. Sci..

[212] Taguchi K., Yamamoto M. (2017). The KEAP1-NRF2 System in Cancer. Front. Oncol..

[213] Obsilova V., Honzejkova K., Obsil T. (2021). Structural Insights Support Targeting ASK1 Kinase for Therapeutic Interventions. Int. J. Mol. Sci..

[214] Nitti M., Marengo B., Furfaro A.L., Pronzato M.A., Marinari U.M., Domenicotti C., Traverso N. (2022). Hormesis and Oxidative Distress: Pathophysiology of Reactive Oxygen Species and the Open Question of Antioxidant Modulation and Supplementation. Antioxidants.

[215] Murphy M.P., Bayir H., Belousov V., Chang C.J., Davies K.J.A., Davies M.J., Dick T.P., Finkel T., Forman H.J., Janssen-Heininger Y. (2022). Guidelines for measuring reactive oxygen species and oxidative damage in cells and in vivo. Nat. Metab..

[216] Lukyanov K.A., Belousov V.V. (2014). Genetically encoded fluorescent redox sensors. Biochim. Biophys. Acta.

[217] Mendoza E.N., Ciriolo M.R., Ciccarone F. (2025). Hypoxia-Induced Reactive Oxygen Species: Their Role in Cancer Resistance and Emerging Therapies to Overcome It. Antioxidants.

[218] Gong S., Wang S., Shao M. (2022). NADPH Oxidase 4: A Potential Therapeutic Target of Malignancy. Front. Cell Dev. Biol..

[219] Li J., Liu J., Zhou Z., Wu R., Chen X., Yu C., Stockwell B., Kroemer G., Kang R., Tang D. (2023). Tumor-Specific GPX4 Degradation Enhances Ferroptosis-Initiated Antitumor Immune Response in Mouse Models of Pancreatic Cancer. Sci. Transl. Med..

[220] Larrauri-Rodríguez K.A., Leon-Chavez B.A., Vallejo-Ruiz V., Peña L.M.P., Maycotte P. (2024). Interplay between reactive oxygen species and ERK activation in cervical cancer cells. Front. Cell Dev. Biol..

[221] Luobin L., Wanxin H., Yingxin G., Qinzhou Z., Zefeng L., Danyang W., Huaqin L. (2024). Nanomedicine-induced programmed cell death in cancer therapy: Mechanisms and perspectives. Cell Death Discov..

[222] Sayin V.I., Ibrahim M.X., Larsson E., Nilsson J.A., Lindahl P., Bergo M.O. (2014). Antioxidants accelerate lung cancer progression in mice. Sci. Transl. Med..

[223] Klein E.A., Thompson I.M., Tangen C.M., Crowley J.J., Lucia M.S., Goodman P.J., Minasian L.M., Ford L.G., Parnes H.L., Gaziano J.M. (2011). Vitamin E and the risk of prostate cancer: The Selenium and Vitamin E Cancer Prevention Trial (SELECT). JAMA.

[224] Griendling K.K., Touyz R.M., Zweier J.L., Dikalov S., Chilian W., Chen Y.R., Harrison D.G., Bhatnagar A. (2016). Measurement of reactive oxygen species, reactive nitrogen species, and redox-dependent signaling in the cardiovascular system. Circ. Res..

[225] Sutton T.R., Minnion M., Barbarino F., Koster G., Fernandez B.O., Cumpstey A.F., Wischmann P., Madhani M., Frenneaux M.P., Postle A.D. (2018). A robust and versatile mass spectrometry platform for comprehensive assessment of the thiol redox metabolome. Redox Biol..

